# Identification of new rice cultivars and resistance loci against rice black-streaked dwarf virus disease through genome-wide association study

**DOI:** 10.1186/s12284-019-0310-1

**Published:** 2019-07-15

**Authors:** Zhiming Feng, Houxiang Kang, Mingyou Li, Lihua Zou, Xiaoqiu Wang, Jianhua Zhao, Lang Wei, Nana Zhou, Qianqian Li, Ying Lan, Yafang Zhang, Zongxiang Chen, Wende Liu, Xuebiao Pan, Guo-Liang Wang, Shimin Zuo

**Affiliations:** 1 Jiangsu Key Laboratory of Crop Genetics and Physiology/ Key Laboratory of Plant Functional Genomics of the Ministry of Education/ Jiangsu Key Laboratory of Crop Genomics and Molecular Breeding, Agricultural College of Yangzhou University, Yangzhou, 225009 China; 2 Jiangsu Co-Innovation Center for Modern Production Technology of Grain Crops, Yangzhou University, Yangzhou, 225009 China; 3 State Key Laboratory for biology of plant diseases and insect pests/Institute of plant protection, Chinese academy of Agricultural Sciences, Beijing, 100093 China; 4 Department of Plant Pathology, the Ohio State University, Columbus, OH 43210 USA; 5 Institute of Plant Protection, Jiangsu Academy of Agricultural Sciences, Nanjing, 210014 China

**Keywords:** Rice (*Oryza sativa* L.), Rice black-streaked dwarf virus (RBSDV) disease, Genome-wide association study (GWAS), Quantitative trait loci (QTLs), Resistance effect

## Abstract

**Background:**

The rice black-streaked dwarf virus (RBSDV) disease causes severe rice yield losses in Eastern China and other East Asian countries. Breeding resistant cultivars is the most economical and effective strategy to control the disease. However, few varieties and QTLs for RBSDV resistance have been identified to date.

**Results:**

In this study, we conducted a genome-wide association study (GWAS) on RBSDV resistance using the rice diversity panel 1 (RDP1) cultivars that were genotyped by a 44,000 high-density single nucleotide polymorphism (SNP) markers array. We found that less than 15% of these cultivars displayed resistance to RBSDV when tested under natural infection conditions at two locations with serious RBSDV occurrence. The *aus*, *indica* and *tropical japonica* sub-populations displayed higher RBSDV resistance than the *aromatic* and *temperate japonica* sub-populations. In particular, we identified four varieties that displayed stable levels of RBSDV resistance at all testing locations. GWAS identified 84 non-redundant SNP loci significantly associated with RBSDV resistance at two locations, leading to the identification of 13 QTLs for RBSDV resistance. Among them, *qRBSDV-4.2* and *qRBSDV-6.3* were detected at both locations, suggesting their resistance stability against environmental influence. Field disease evaluations showed that *qRBSDV-6.3* significantly reduces RBSDV disease severity by 20%. Furthermore, introgression of *qRBSDV-6.3* into two susceptible rice cultivars by marker-assisted selection demonstrated the effectiveness of *qRBSDV-6.3* in enhancing RBSDV resistance.

**Conclusions:**

The new resistant cultivars and QTLs against RBSDV disease identified in this study provide important information and genetic materials for the cloning of RBSDV resistance genes as well as developing RBSDV resistant varieties through marker-assisted selection.

**Electronic supplementary material:**

The online version of this article (10.1186/s12284-019-0310-1) contains supplementary material, which is available to authorized users.

## Background

The rice black-streaked dwarf virus (RBSDV) disease is spread by small brown planthoppers (*Laodelphax striatellus* Fallén, SBPH) (Shikata and Kitagawa 1977; Ruan et al. 1984). Typical symptoms of the RBSDV disease include severe dwarf shrinkage, dark-green and rigid leaves, and short white waxy or black-streaked stripes along the abaxial leaf surface and veins on the stem (Ruan et al. 1984; Zhou et al. 2008; Zhou 2013). Once the plants are infected, it is generally incurable; therefore, the RBSDV disease is also known as “cancer” in rice (Azuhata et al. 1993). In recent years, the RBSDV disease is expanding in Eastern China and other East Asian countries, resulting in severe yield losses (Sun et al. 2013; Zhou et al. 2015). Breeding resistant cultivars are believed to be the more economical and effective strategies to control the RBSDV disease compared to pesticides spraying to control the transmission vector SBPH (Zhou et al. 2015; Sun et al. 2017). However, only a few studies reported the mapping of resistance genes and quantitative trait loci (QTLs) to RBSDV to date (Pan et al. 2009; Wang et al. 2010; Zheng et al. 2012; Li et al. 2013; Zhou et al. 2015; Zhang et al. 2016; Sun et al. 2017).

The resistance of rice to RBSDV has been reported to be a quantitative trait controlled by QTLs or multiple genes (Pan et al. 2009; Wang et al. 2010; Zheng et al. 2012). Using recombinant inbred lines (RILs) derived from a cross between Koshihikari and Guichao2, Wang et al. (2010) detected a QTL for RBSDV resistance on chromosome 3 with resistant alleles from Koshihikari. Li et al. (2013) identified 3 QTLs for RBSDV resistance on chromosomes 6, 7, and 9 with resistant alleles from Minghui63 using a RIL population derived from a Zhenshan97/Minghui63 cross; the major QTL on chromosome 6 was further located at a 627.6-kb interval. The resistance of the Tetep cultivar to RBSDV is controlled by 3 QTLs located on chromosomes 3, 10, and 11, respectively (Zhou et al. 2015). Recently, several more QTLs for RBSDV resistance were identified on chromosomes 1, 6, 8, and 9 using a RIL population derived from a cross between IR36 and L5494 (Zhang et al. 2016). Sun et al. (2017) identified a highly resistant variety 9194 and mapped 4 QTLs for RBSDV resistance on chromosomes 3, 6, 9, and 11 using an F_2:3_ population derived from a cross between 9194 and Suyunuo. In general, due to the lack of highly resistant resources and major QTLs for RBSDV resistance, the progress of genetic study and breeding of rice resistance to RBSDV is slow. Therefore, in order to breed resistant cultivars and reduce yield losses caused by RBSDV, it is urgent to identify highly resistant germplasm and to map major QTLs for RBSDV resistance using new mapping techniques.

With the advancement of sequencing technology, genome-wide association study (GWAS) using high-density genome-wide single nucleotide polymorphisms (SNPs), has become a powerful and popular strategy for mining genes/QTLs controlling complex traits in plants (Buckler et al. 2009; Huang et al. 2010; Brachi et al. 2011; Zhao et al. 2011; Morris et al. 2013; Wang et al. 2016; Yano et al. 2016; Li et al. 2017). Compared to the traditional genetic linkage method that requires bi-parental mapping populations, GWAS is based on diverse natural populations and can detect multiple variants at an identified locus (Flint-Garcia et al. 2003). In rice, many genes/QTLs related to growth, development, biotic and abiotic stress tolerance have been detected by GWAS (Huang et al. 2010; Famoso et al. 2011; Zhao et al. 2011; Kang et al. 2016; Zhu et al. 2016; Li et al. 2017). The identification of allelic variations in phenotypic diversity germplasm collections will be of great practical significance to rice breeding. However, there is no report for the identification of QTLs associated with RBSDV resistance by GWAS.

The rice diversity panel 1 (RDP1), which consists of about 420 *Oryza sativa* accessions from 82 countries, is divided into six sub-populations (*tropical japonica* [TRJ], *temperate japonica* [TEJ], *indica* [IND], *aus* [AUS], *aromatic* [ARO]) and admixture [ADM]) by structure analysis, and contains a large number of phenotypic and genetic diversity (Zhao et al. 2011; Eizenga et al. 2014; Zhu et al. 2016). Importantly, the RDP1 was genotyped with about 44,000 high-quality SNPs, which provides the basis for GWAS (McCouch et al. 2010; Tung et al. 2010). Recently, the RDP1 has been used to identify many genes/QTLs related to diverse traits in rice, such as heading date, protein content, panicle number, seed number per panicle, blast resistance, sheath blight resistance, aluminum and ozone tolerance (Famoso et al. 2011; Zhao et al. 2011; Norton et al. 2014; Ueda et al. 2015; Kang et al. 2016; Chen et al. 2019).

In this study, the RBSDV resistance of the RDP1 cultivars were evaluated in the fields and then GWAS was used to identify the RBSDV QTLs in the rice genome. Several varieties with high RBSDV resistance levels and 13 QTLs for RBSDV resistance were identified. Our results will be useful for the identification of candidate genes controlling RBSDV resistance as well as developing RBSDV resistant rice varieties through marker-assisted selection (MAS).

## Results

### Less than 15% of the RDP1 varieties showed partial resistance to RBSDV with disease incidence lower than 20%

**Fig. 1:**
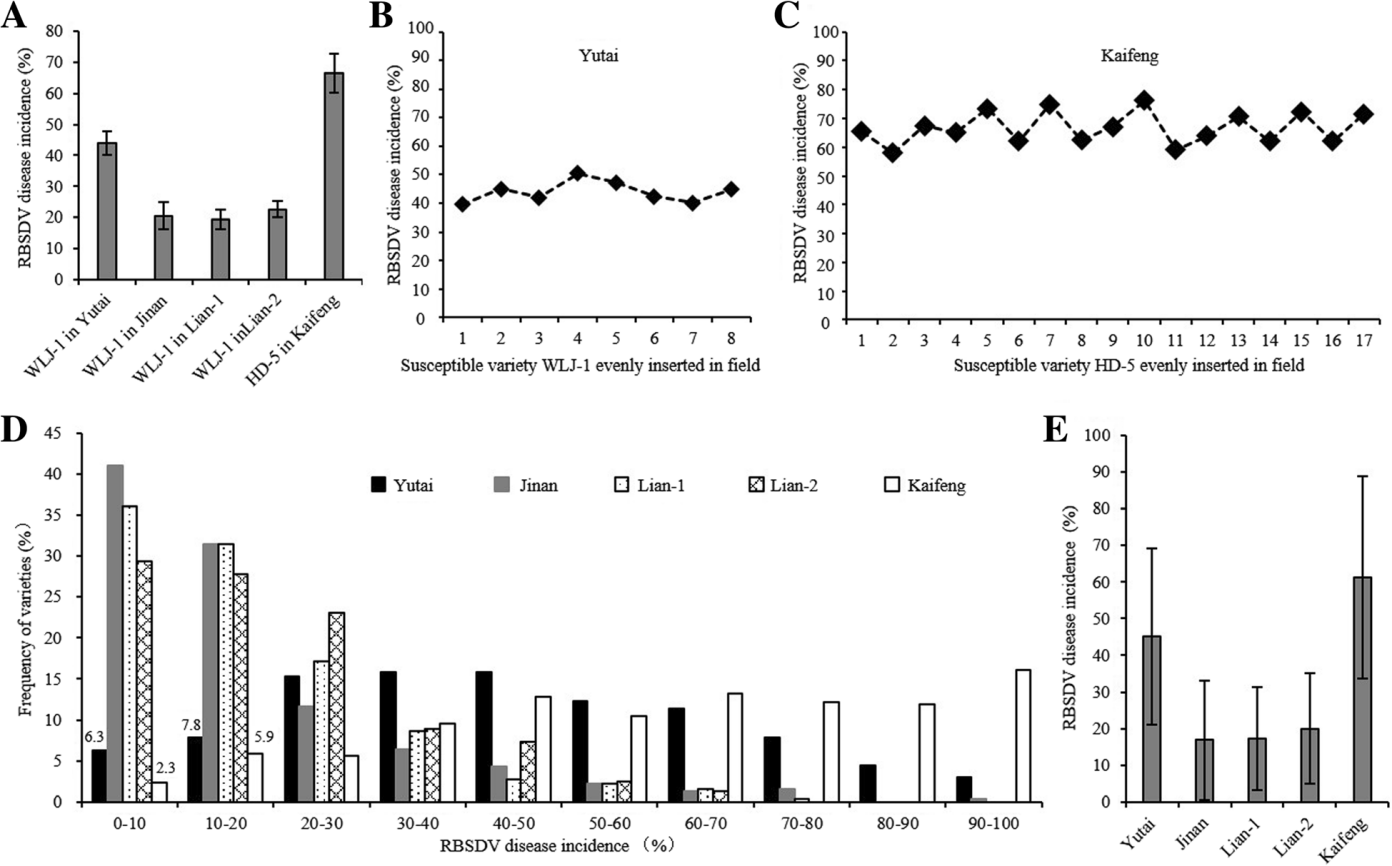
Phenotypes of the RDP1 and the susceptible control for the RBSDV disease at different locations. **a** The RBSDV disease incidences of the susceptible controls, WLJ-1 and HD-5. **b** and **c** RBSDV disease incidences of susceptible controls planted in different parts of the field in Yutai (**b**) and Kaifeng (**c**). **d** Frequency distributions of varieties with different disease incidences of rice at different locations. **e** Average RBSDV disease incidences of all varieties at each location

A total of 305 rice cultivars of the RDP1 and the two susceptible control cultivars were evaluated for RBSDV resistance after natural infection in Yutai County and Jinan City in Shandong Province (2013), Lian Yungang City (two testing locations, called lian-1 and lian-2) in Jiangsu Province (2013), and Kaifeng City in Henan Province (2014). The disease incidences (DIs) of the RBSDV disease and their frequency distributions for these cultivars are shown in Additional file 1: Table S1 and Fig. 1. The DIs of the susceptible control WLJ-1 were 20.5%, 19.2% and 22.6% at Jinan, Lian-1 and Lian-2, respectively, while, those of WLJ-1 and HD-5 were 43.9% and 66.5% at Yutai and Kaifeng, respectively (Fig. 1a), indicating that the RBSDV disease at Yutai and Kaifeng was more prevalent than at Jinan, Lian-1 and Lian-2. We further compared the DIs of the susceptible controls at different positions in the fields in Yutai and Kaifeng, and found that the DIs were 39.5~50.5% for WLJ-1 and 62%~ 72% for HD-5 (Fig. 1 b and c), indicating that varieties at the different positions could receive similar doses of RBSDV. Furthermore, the DIs of the RDP1 varieties were mostly within 30% with average DIs between 10%–20% at Jinan, Lian-1 and Lian-2, while mostly between 20%~ 70% and 40%~ 100% with average DIs of 43.1% and 61.3% at Yutai and Kaifeng, respectively (Fig. 1d and e). This result confirmed that the RBSDV disease occurrence rates at Yutai and Kaifeng were higher than those at Jinan, Lian-1 and Lian-2. Taken together, we concluded that the DIs data from Yutai and Kaifeng were more suitable for further screening of resistant germplasm and identifying RBSDV resistance genes.

Based on the DIs data at the Yutai and Kaifeng locations, we found that most of the tested varieties were susceptible to RBSDV, that no completely immune varieties were detected, and that only 14.1% (Yutai) and 8.2% (Kaifeng) of the tested varieties displayed partial resistance to RBSDV with a DI lower than 20% (Fig. 1; Additional file 1: Table S1), suggesting that resistance to RBSDV of most varieties was minimal.

### Four varieties were identified with stable resistance to RBSDV

**Fig. 2:**
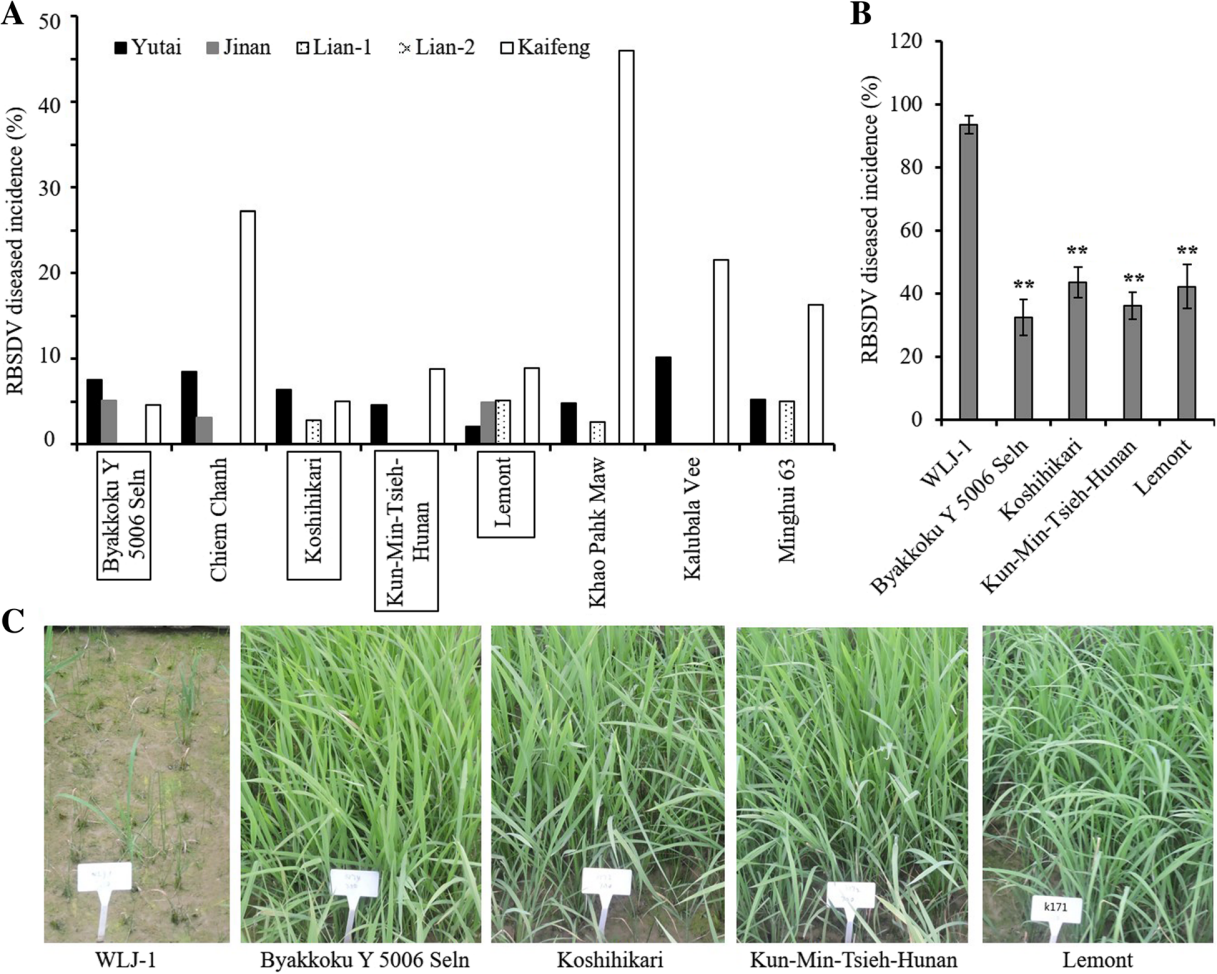
Phenotypes of 4 identified RBSDV resistant varieties. **a** Phenotype comparison of the candidate RBSDV-resistance varieties among 5 locations. Rectangles highlight the newly identified resistant varieties with stable RBSDV resistance level. **b** and **c** Disease incidences (**b**) and images (**c**) of 4 RBSDV-resistance varieties and WLJ-1 control after artificial inoculation with RBSDV. **P < 0.01 by Student’s t-test

From the DI data of all varieties evaluated at Yutai, Jinan, Lian-1 and Lian-2 in 2013, we identified 8 candidate resistant varieties with DI less than 10%, including the previously reported resistant variety Minghui 63 (Fig. 2a; Li et al. 2013). These 8 varieties were further evaluated at Kaifeng in 2014; the DI of 4 of them (Byakkoku Y5006 SelN, Koshihikari, Kun-Min-Tsieh-Huran and Lemont) were lower than 10% while the remaining 4 (including Minghui 63) exceeded 10% (Fig. 2a). Artificial inoculation was performed under controlled conditions to further validate the RBSDV resistance of the 4 resistant varieties. Compared to the susceptible control WLJ-1 yielded a DI of 93.6 ± 2.9%, these 4 varieties showed DI values of 32.4 ± 5.6%, 43.5 ± 4.9%, 36.0 ± 4.3% and 42.2 ± 6.9%, respectively (Fig. 2 b and c), which are consistent with the field results. Collectively, we infer that the 4 varieties confer relatively high and stable resistance to RBSDV. Among them, we found Koshihikari and Lemont have been reported by the previous studies (Wang et al. 2010; Zheng et al. 2012), and the remaining two varieties, Byakkoku Y5006 SelN and Kun-Min-Tsieh-Huran, are newly identified in our study.

The Kaifeng location showed the most serious RBSDV occurrence as well as SBPH infestation in 2014 (Sun et al. 2017), presenting a good opportunity for us to determine whether the RBSDV resistance levels correlate with their resistance levels to SBPH, which transmits RBSDV. After preliminary observation by visual, we found that 4 of all plots/varieties apparently contained more SBPH than the other plots, and 7 plots/varieties contained less SBPH. For further quantifying the SBPH density, we counted the number of SBPH in these 11 varieties and other 17 varieties randomly selected (Table 1). We found that the 4 varieties did contain high density of SBPH with more than 20 SBPH per plant, while the 7 varieties did harbor low density of SBPH with no more than 3 SBPH per plant. The remaining 17 varieties, including the new RBSDV resistant variety Byakkoku Y5006 SelN, carried the number of SBPH range from 13 to 17; (Table 1). These suggest that most varieties showed similar response to SBPH, while the 4 varieties were probably susceptible to SBPH and the other 7 had SBPH resistance although the resistant mechanism is unclear yet.

**Table 1:** Disease incidences of the RBSDV disease and the number of SBPH landing on the varieties

Accession Name	Country/region of origin	Sub-population	Number of SBPH (mean ± SD)	RBSDV disease incidence at different locations	
				Kaifeng	Yutai
Bulgare	France	TEJ	25.3 ± 5.8	73.7%	11.5%
DZ 193	Bangladesh	AUS	20.8 ± 2.4	96.8%	77.8%
NPE 835	Pakistan	TEJ	22.8 ± 4.2	96.8%	77.8%
Ta Mao Tsao	China	TEJ	23.6 ± 3.6	92.3%	85.7%
Average DI of varieties with high SBPH density				89.9% ± 11.0%^a^	63.2% ± 34.7%
Gerdeh	Iran	ADM	16.6 ± 3.1	96.0%	62.5%
Jouiku 393G	Japan	TEJ	15.1 ± 2.3	89.9%	37.5%
LAC 23	Liberia	TRJ	18.4 ± 4.6	51.9%	44.4%
Nova	United States	ADM	13.9 ± 1.5	58.8%	8.3%
Romeo	Italy	TEJ	15.2 ± 3.3	91.9%	80.0%
Byakkoku Y 5006 Seln	Australia	IND	14.3 ± 3.6	4.5%	7.5%
Bergreis	Austria	TEJ	16.7 ± 5.2	100.0%	–
Azerbaidjanica	Azerbaijan	TEJ	13.3 ± 2.9	100.0%	100.0%
Karabaschak	Bulgaria	TEJ	15.8 ± 4.4	100.0%	85.7%
M. Blatec	Macedonia	ADM	14.1 ± 1.9	97.5%	100.0%
Triomphe Du Maroc	Morocco	TEJ	13.0 ± 2.5	82.1%	60.0%
Lusitano	Portugal	TEJ	15.8 ± 4.3	96.3%	25.0%
WIR 3764	Uzbekistan	TEJ	13.7 ± 3.3	94.9%	66.7%
Okshitmayin	Myanmar	ADM	14.3 ± 4.1	83.5%	78.9%
Sanbyang-Daeme	Korea	ADM	15.2 ± 2.7	67.9%	66.7%
Heukgyeong	South Korea	TEJ	17.4 ± 4.6	100.0%	67.7%
Bengal	United States	ADM	16.8 ± 3.9	44.3%	64.3%
Average DI of varieties with moderate SBPH density				80.0% ± 26.5%^bc^	59.7% ± 28.51%
LD 24	Sri Lanka	IND	1.7 ± 0.8	30.0%	7.4%
Dee Geo Woo Gen	Taiwan	IND	1.9 ± 1.2	81.5%	69.8%
Karkati 87	Bangladesh	AUS	2.1 ± 0.8	86.5%	50.0%
Chibica	Mozambique	TEJ	2.8 ± 1.5	61.3%	48.2%
Kiang-Chou-Chiu	Taiwan	IND	2.0 ± 1.8	63.8%	21.8%
Shangyu 394	China	TEJ	2.4 ± 1.1	68.4%	62.5%
Aijiaonante	China	IND	1.5 ± 1.3	82.3%	84.2%
Average DI of varieties with low SBPH density				67.7% ± 19.3%^b^	49.1% ± 26.9%

Different small letters indicate the difference on 5% statistically significant difference. ADM, Admixture; AUS, *aus*; IND, *indica*; ARO, *aromatic*; TEJ, *temperate japonica*; TRJ, *tropical japonica*

We further compared the DIs of the varieties with high, moderate and low density of SBPH, and found that at Kaifeng location the varieties with low density of SBPH carried significantly stronger RBSDV resistance than those with high density of SBPH; while, no significant differences were found between varieties with high and moderate density of SBPH (Table 1). In addition, three varieties with low density of SBPH showed high DIs (by more than 80%), and there were no significant differences on RBSDV DIs of the three types of varieties at Yutai location (Table 1). These implied that the SBPH density is not the critical factor on affecting variety resistance to RBSDV under the condition of serious SBPH and RBSDV occurrences in field. The 8 candidate RBSDV resistance varieties identified in 2013 contained no less than 6 SBPH per plant (Additional file 2: Table S2), closing to that of the 17 varieties with moderate SBPH density. Except the Byakkoku Y5006 SelN, the 3 RBSDV resistant varieties, Koshihkari, Kun-Min-Tsieh-Huran and Lemont, confirmed in 2017 were found with 6.8 ± 1.8, 9.3 ± 2.5 and 8.4 ± 1.3 SBPH, respectively. Taken together, considering the fact that the 4 RBSDV resistant varieties did not belong to the type of low SBPH density, we speculate that they most likely have ability to counter RBSDV but not SBPH, and would be useful for the identification of RBSDV resistance genes.

### *Indica* varieties carry the highest RBSDV resistance level among the six rice sub-populations

**Fig. 3:**
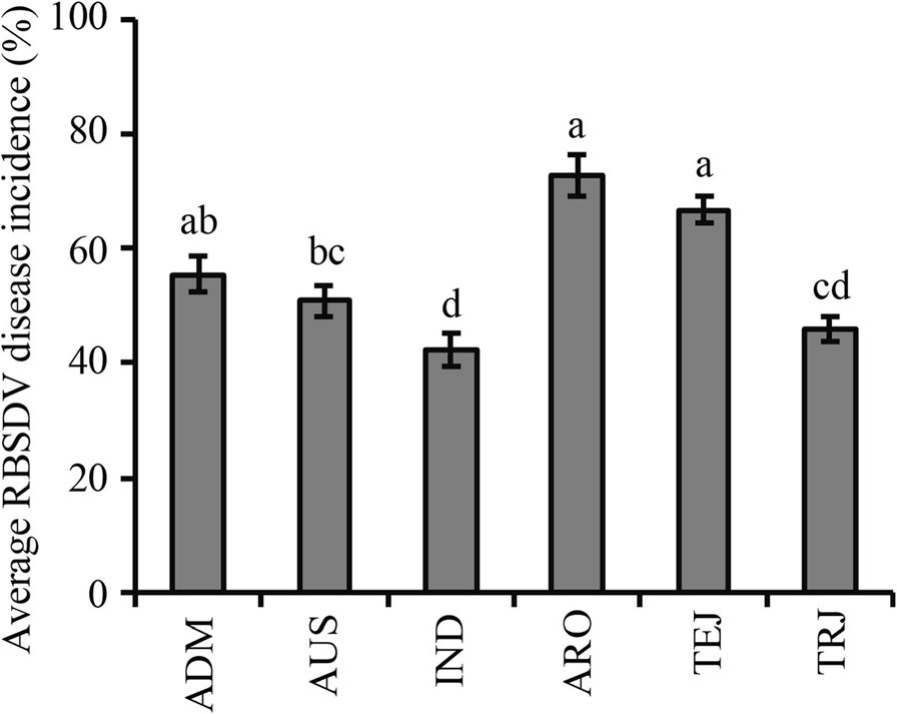
Multi-comparison of RBSDV disease incidences of different sub-populations in the RDP1. Letters above columns indicate different statistical groups on 5% statistically significant difference. ADM, Admixture; AUS, *aus*; IND, *indica*; ARO, *aromatic*; TEJ, *temperate japonica*; TRJ, *tropical japonica*

To understand the differences in RBSDV resistance among sub-populations of the RDP1 cultivars, we analyzed the RBSDV resistance of the six rice sub-populations reported previously (Zhu et al. 2016). The analysis result showed that the AUS (average DI was 50.9% between Yutai and Kaifeng), IND (42.4%) and TRJ (45.9%) sub-populations were significantly more resistant than the ARO (72.8%) and TEJ (66.8%) sub-populations (Fig. 3). What’s more, the IND sub-population carries the highest overall RBSDV resistance level among the six rice sub-populations.

### Both novel and previously identified QTLs associated with RBSDV resistance were detected

**Fig. 4:**
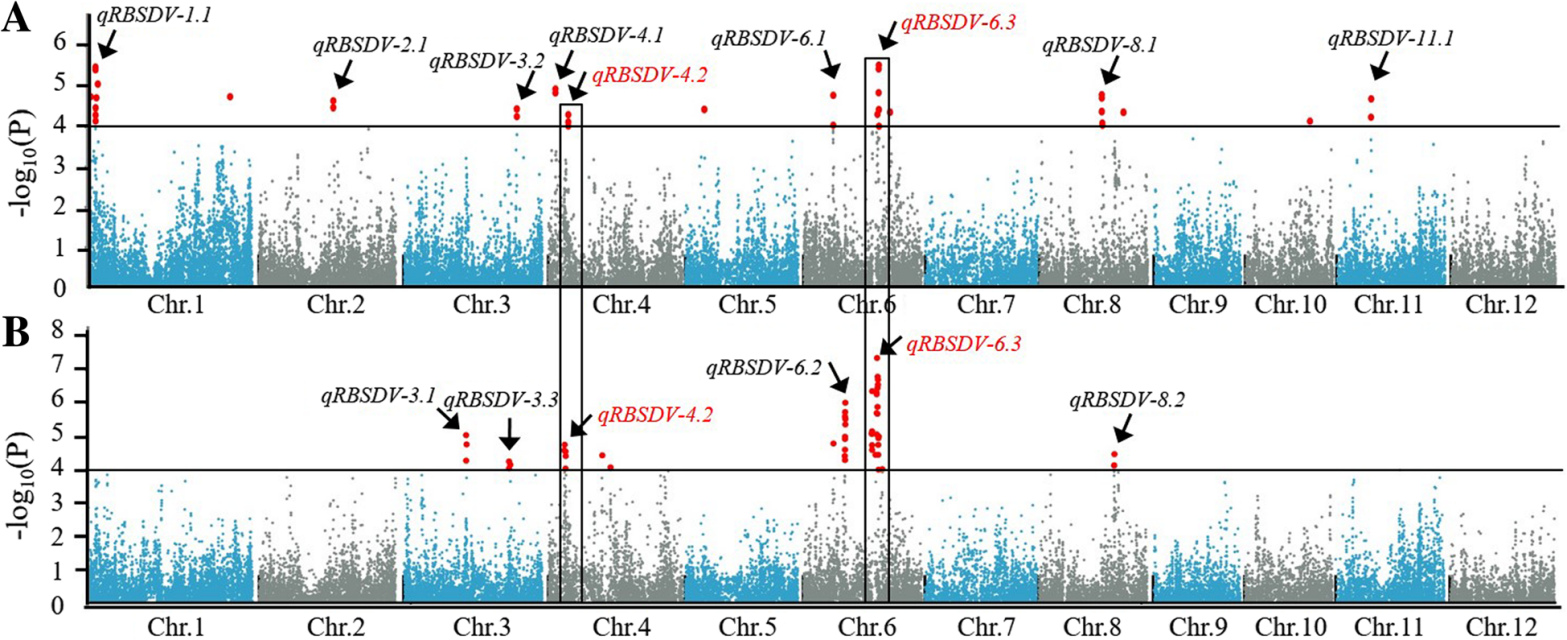
Genome-wide association study of RBSDV resistance of 305 rice varieties. **a** and **b** Manhattan plots of MLM for the data obtained at Kaifeng (**a**) and Yutai (**b**). Black horizontal lines indicate the genome-wide significance threshold. Arrows indicate regions containing at least 2 significant-association markers which were designed as QTLs for RBSDV resistance. The two regions highlighted by rectangles indicate they were detected at both locations with consistent results

To identify loci associated with RBSDV resistance, GWASs were carried out using the 44-K SNP data set and DI data of the RDP1 cultivars obtained from the Kaifeng and Yutai locations. Total of 35 and 54 SNP loci that were significantly associated with RBSDV resistance were found from the Kaifeng and Yutai test, repectively. The contribution of each significant association marker on phenotypic variance ranged from 5.03% to 9.94% (Fig. 4; Additional file 3: Table S3). These SNP loci were mainly distributed on chromosomes 1, 2, 3, 4, 6, 8 and 11 (Fig. 4). Among them, 9 and 6 associated regions that had at least 2 significant association markers from the Kaifeng and Yutai test were identified, respectively (Fig. 4). We designated these QTLs as *qRBSDV-1.1*, *qRBSDV-2.1*, *qRBSDV-3.1*, *qRBSDV-3.2*, *qRBSDV-3.3*, *qRBSDV-4.1*, *qRBSDV-4.2*, *qRBSDV-6.1*, *qRBSDV-6.2*, *qRBSDV-6.3*, *qRBSDV-8.1*, *qRBSDV-8.2*, and *qRBSDV-11.1*, respectively (Fig. 4; Table 2). Most significantly, *qRBSDV-4.2* and *qRBSDV-6.3* were detected at both locations (Fig. 4), suggesting that these two QTLs were stable against the environmental influence. By comparing with the previously reported RBSDV QTLs, we found that *qRBSDV-3.2* and *qRBSDV-3.3* are collocated with *qRBSDV3b* (Zheng et al. 2012), *qRBSDV-11.1* collocated with *qRBSDV-11* (Zhou et al. 2015), *qRBSDV-6.3* partially overlapped with *qRBSDV6* detected by Sun et al. (2017), and the remaining 9 QTLs were not reported previously (Table 2).

**Table 2:** QTLs for the RBSDV disease

QTL	Chromsome	Position	Top SNP marker	Loci reported^a^	Reference
*qRBSDV-1.1*	1	2098423–2727493	id1001680		
*qRBSDV-2.1*	2	19545113–29737544	id2007622		
*qRBSDV-3.1*	3	16421475–16550808	id3008188		
*qRBSDV-3.2*	3	29724621–29737544	ud3001634	*qRBSDV3b*	Zheng et al. 2012
*qRBSDV-3.3*	3	27699444–28028330	id3012227	*qRBSDV3b*	Zheng et al. 2012
*qRBSDV-4.1*	4	1875972–1876773	id4000919		
*qRBSDV-4.2*	4	4172738–5255769	id4001777		
*qRBSDV-6.1*	6	7835229–7840016	id6004955		
*qRBSDV-6.2*	6	10959905–11106570	id6007011		
*qRBSDV-6.3*	6	17993684–19865608	id6010277	*qRBSDV6*	Sun et al. 2017
*qRBSDV-8.1*	8	16332728–16524733	id8004300		
*qRBSDV-8.2*	8	19756382–19826236	id8005332		
*qRBSDV-11.1*	11	9028442–9075159	id11003528	*qRBSDV-11*	Zhou et al. 2015

^a^QTLs reported to confer resistance to RBSDV

*qRBSDV-6.3* was detected with more association markers than others and is located in a region between SNPs id6010063 and id6010534 covering 1.9 Mb (Additional file 3: Table S3; Table 2). In this region, five significant association markers (id6010277, id6010459, id6010472, id6010489 and id6010523) that covered a 439.154 Kb region were repeatedly detected at both test locations (Fig. 4; Additional file 3: Table S3), indicating that *qRBSDV-6.3* is likely located within or near the interval of these 5 significant association markers. We identified 32 candidate genes in this 439.154 Kb region (Additional file 4: Table S4). Those candidate genes were classified into 17 category based on their annotation (Additional file 7: Figure S1). The four major categories were hypothetical protein (12), abscisic acid receptor PYL3-like (3), probable protein phosphatase 2C (2), and cyclic nucleotide-gated ion channel (2). For *qRBSDV-4.2*, although the most significant association markers (id4001630 and ud4000384, respectively) that detected were different at the two test locations, the distance between the two markers was about 1 MB (Additional file 3: Table S3), implying that *qRBSDV-4.2* is likely located within this 1 MB region. In addition, *qRBSDV-1.1* and *qRBSDV-6.2* were also identified with higher degree of association (Fig. 4).

### *qRBSDV-6.3* has obvious effects on reducing RBSDV disease severity

To further assess the effect of *qRBSDV-6.3* on RBSDV resistance, we employed the 5 stable significant association SNPs in the *qRBSDV-6.3* region to distinguish varieties with and without the resistant alleles. The favorable/unfavorable alleles of these 5 SNPs (id6010277, id6010459, id6010472, id6010489 and id6010523) were A/C, T/A, C/T, G/A, and T/G, respectively. Their distributions are generally balanced in the RDP1 (Table 3). We found that the favorable allele of each SNP reduced the DI value by more than 15% at both test locations based on the relevant statistical parameters in the GWASs and the DI data between the favorable and unfavorable allele of each SNP (Table 3).

**Table 3:** Statistic information of the five significant association SNP markers in Kaifeng and Yutai locations

Marker	Site	Allele	Kaifeng			Yutai			Average		
			Effect (%)	Obs	Marker R^2^	Effect (%)	Obs	Marker R^2^	Effect (%)	Obs	Marker R^2^
id6010277	19426454	A	−16.63	142	5.44	−19.66	137	9.94	−16.93	142	8.73
		C	0.00	155		0.00	152		0.00	155	
id6010459	19737177	T	−17.82	151	6.04	−15.17	146	5.66	−15.36	151	6.96
		A	0.00	153		0.00	150		0.00	153	
id6010472	19739735	C	−19.21	142	6.94	−15.90	137	6.16	−16.24	142	7.73
		T	0.00	158		0.00	155		0.00	158	
id6010489	19785959	G	−19.32	144	6.98	−16.22	139	6.36	−16.48	144	7.89
		A	0.00	160		0.00	157		0.00	160	
id6010523	19827029	T	−17.52	150	4.92	−15.38	144	5.03	−15.50	150	6.02
		G	0.00	154		0.00	152		0.00	154	

*Obs* observation

**Fig. 5:**
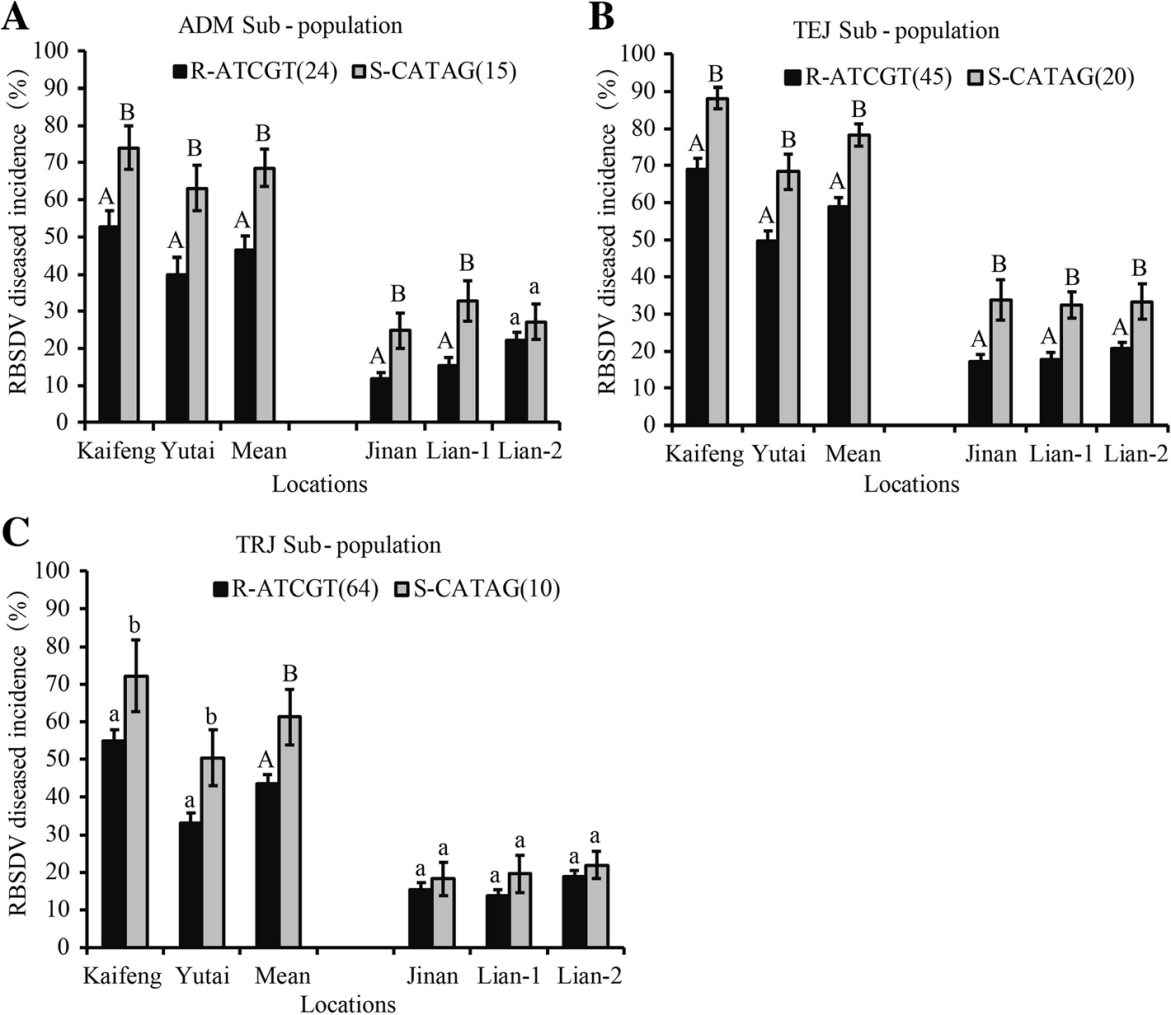
Resistance comparison of the varieties with different haplotypes formed by 5 significant association SNP markers. a-c Resistance comparison of different haplotypes in ADM (**a**), TEJ (**b**) and TRJ (**c**) sub-populations. The haplotypes were formed from the 5 significant association SNP markers in the order of id6010277, id6010459, id6010472, id6010489 and id6010523 on chromosome 6. R-ATCGT and S-CATAG indicate resistant (favorable) and susceptible (unfavorable) haplotypes, respectively. The number following R-ATCGT or S-CATAG indicates the number of varieties having this haplotype. Different capital and lowercase letters above columns indicate 1% and 5% statistically significant difference, respectively. RBSDV, rice black-streaked dwarf virus; ADM, Admixture; TEJ, *temperate japonica*; TRJ, *tropical japonica*

Further analysis showed that when the 5 SNP markers were combined there were 7 haplotypes in the RDP1 varieties, with the favorable haplotype being ‘ATCGT’ and the unfavorable being ‘CATAG’ (Additional file 5: Table S5). The number of varieties was 133 for the ‘ATCGT’ haplotype, 138 for ‘CATAG’, and 8 or less for each of the other 5 haplotypes (Additional file 5: Table S5). We found that there was no significant difference in the RBSDV resistance level between ‘ATCGT’ and ‘CATAG’ haplotypes (Additional file 5: Table S5). To test whether this might have resulted from population structure, we first examined what rice sub-populations each haplotype contained and then compared their RBSDV resistance levels. The unfavorable ‘CATAG’ haplotype contained AUS, IND, TEJ, TRJ and ADM sub-populations, while the favorable ‘ATCGT’ haplotype only contained ADM, TEJ and TRJ sub-populations but not AUS or IND sub-populations. These results implied that there exists a difference in the population structure of the RDP1 varieties between these two haplotypes. Thus, we were only able to examine among the ADM, TEJ and TRJ sub-populations. We found that among these three sub-populations the favorable ‘ATCGT’ varieties displayed significantly, or even extremely significantly, lower average DIs than the unfavorable ‘CATAG’ varieties under all five locations, and the favorable ‘ATCGT’ haplotype reduced RBSDV disease severity by about 20% under heavy RBSDV pressure at Kaifeng (Fig. 5). These results suggest that *qRBSDV-6.3* had an obvious effect on RBSDV resistance.

### Introgression of *qRBSDV-6.3* into susceptible rice cultivars through MAS improves their RBSDV resistance

**Fig. 6:**
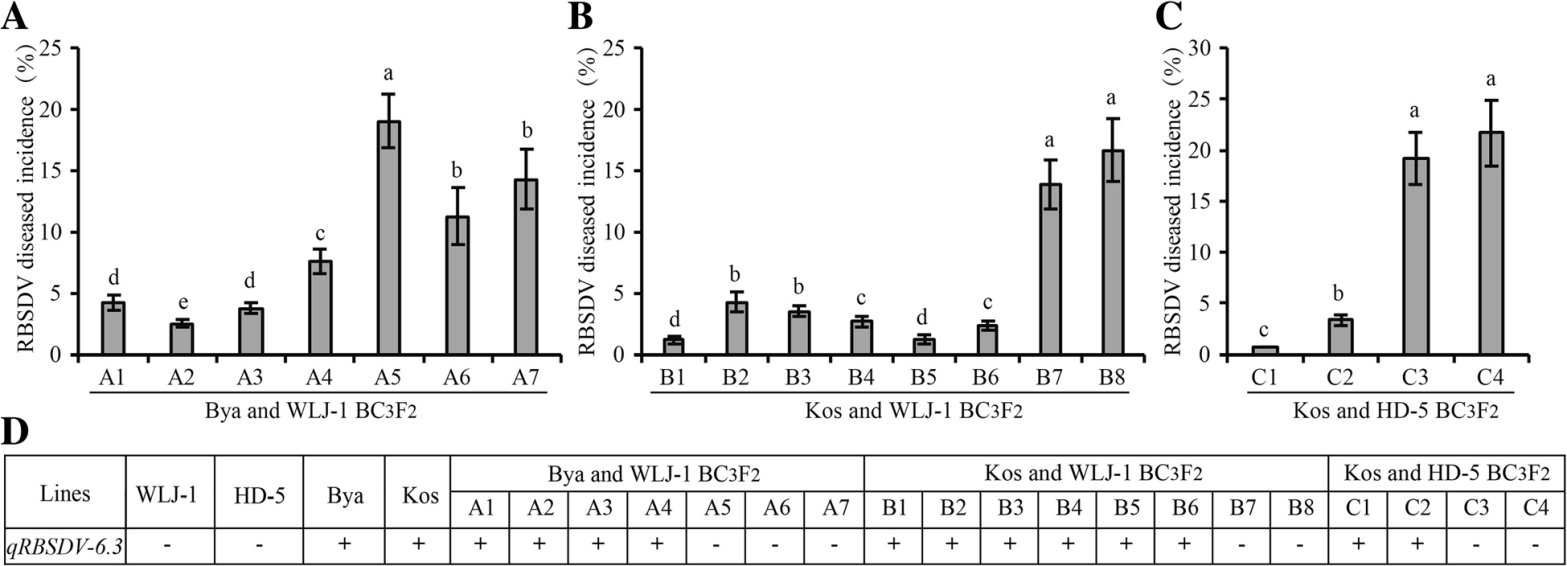
The RBSDV resistance of introgressed homozygous lines containing *qRBSDV-6.3* in three BC_3_F_2_ populations. **a** and **b** The resistance incidences of different lines from BC_3_F_2_ populations by the backcross between Bya (**a**) or Kos (**b**) with WLJ-1, using Kos as the recurrent parent. **c** The resistance incidences of different lines from a BC_3_F_2_ population by the backcross between Kos with HD-5, using Kos as the recurrent parent. **d** The presence (+) or absence (-) of qRBSDV-6.3 in different lines from three BC_3_F_2_populations. Bya, Byakkoku Y 5006 Seln; Kos, Koshihikari. Letters above columns indicate 5% statistically significant difference

To validate the usability of *qRBSDV-6.3*, we introduced *qRBSDV-6.3* from moderately resistant varieties Byakkoku Y 5006 Seln and Koshihikari into highly susceptible varieties WLJ-1 and HD-5. MAS was used to facilitate the development of three BC_3_F_2_ populations: a Byakkoku Y 5006 Seln x WLJ-1 BC_3_F_2_ population, a Koshihikari x WLJ-1 BC_3_F_2_ population, and a Koshihikari x HD-5 BC_3_F_2_ population, using WLJ-1 or HD-5 as the recurrent parent. We obtained a total of 12 *qRBSDV-6.3* introgressed homozygous lines and 7 homozygous lines without the *qRBSDV-6.3* allele from the three BC_3_F_2_ populations and evaluated them for RBSDV resistance. As shown in Fig. 6, the *qRBSDV-6.3* positive lines clearly all displayed markedly higher resistance levels than the *qRBSDV-6.3* negative lines in all three BC_3_F_2_ populations, reducing their DIs roughly from 15 to 20% (*qRBSDV-6.3* negative) to 2–5% (*qRBSDV-6.3* positive). Taken together, these data further confirm that *qRBSDV-6.3* is a reliable QTL against RBSDV and can be employed to improve rice RBSDV resistance.

## Discussion

In recent years, the rice RBSDV disease has become such a serious rice disease that it has attracted great attention. Because RBSDV is not transmitted to plant offspring through the ovary, artificial infection by RBSDV using SBPH carrying RBSDV presents a great challenge, which leads to a serious lag in the research on identifying genetic resistance to RBSDV (Zhou 2013). So far, there are only a few reports about QTLs for RBSDV that all relied on natural infection in the field (Pan et al. 2009; Wang et al. 2010; Zheng et al. 2012; Li et al. 2013; Zhou et al. 2015; Zhang et al. 2016; Sun et al. 2017). In this study, we evaluated the RDP1 cultivars for resistance to RBSDV using the natural infection method at five testing locations where the RBSDV disease was prevalent in the previous years (Zhou 2013). Among these five testing locations, Kaifeng had the highest disease rate with the DI of the susceptible control HD-5 reaching 66.5%, which is close to the DI of the artificial infection method (Zhou et al. 2015; Additional file 1: Table S1; Fig. 1). In addition, the DIs of the susceptible control WLJ-1 and HD-5 at different positions of the field in Yutai and Kaifeng were similar, showing that varieties at the different positions in the field received similar doses of RBSDV (Fig. 1). Therefore, the natural infection by RBSDV at Yutai and Kaifeng were successful, laying the foundation for our successful screening for resistant cultivars and for further genetic research of RBSDV resistance.

We identified 4 RBSDV-resistant cultivars (Byakkoku Y5006 SelN, Koshihikari, Kun-Min-Tsieh-Huran and Lemont) through screens based on natural infection (Fig. 2). To exclude those caused by insect resistance, we investigated the number of SBPH at the seedling stage, and found that the SBPH density did not significantly affect the RBSDV resistance phenotype (Table 1) and the 4 RBSDV-resistant varieties did not belong to the type of low SBPH density. So we speculate that the RBSDV resistances of them were most likely not dependent on resistance to SBPH and thus they most likely contain novel genes/alleles that confer resistance to RBSDV employing unexplored mechanisms. In the future study, we will evaluate the SBPH resistance level of them in the laboratory condition using the standard method including antibiosis test and non-preference test to verify our speculation.

To date, there are no reports dissecting the genetic architecture of resistance to RBSDV by using GWAS strategy. Here we identified 84 non-redundant SNP markers significantly associated with RBSDV resistance through GWAS with high density SNP markers, leading to the identification of 13 QTLs for RBSDV resistance (Fig. 4; Additional file 3: Table S3). Previously reported loci using traditional bi-parents QTL mapping strategy for RBSDV resistance were distributed on chromosomes 1, 3, 4, 6, 7, 8, 9, and 11 (Pan et al. 2009; Wang et al. 2010; Zheng et al. 2012; Li et al. 2013; Zhou et al. 2015; Zhang et al. 2016; Sun et al. 2017). By comparing the physical locations of them with 13 QTLs identified in our study, we found that *qRBSDV-1.1*, *qRBSDV-2.1*, *qRBSDV-2.1*, *qRBSDV-3.1*, *qRBSDV-4.1*, *qRBSDV-4.2*, *qRBSDV-6.1*, *qRBSDV-6.2*, *qRBSDV-8.1*, and *qRBSDV-8.2* did not locate to regions with known RBSDV resistance loci (Table 2), suggesting that these 9 QTLs are novel loci related to RBSDV resistance. Although *qRBSDV-3.2* and *qRBSDV-3.3* are collocated with *qRBSDV3b* detected by Zheng et al. (2012), *qRBSDV-11.1* collocated with *qRBSDV-11* detected by Zhou et al. (2015), and *qRBSDV-6.3* partially overlapped with *qRBSDV6* detected by Sun et al. (2017), these 4 QTLs are likely not controlled by the same genes or alleles as their corresponding reported RBSDV QTLs.

*qRBSDV-6.3* was identified with the highest number of association SNP markers and degree of association among all regions (Fig. 4; Additional file 3: Table S3), and was estimated to reduce RBSDV disease severity by approximately 20% under severe RBSDV conditions (Fig. 5). Furthermore, when introgressed into susceptible rice cultivars through MAS, *qRBSDV-6.3* significantly increased RBSDV resistance in all three BC_3_F_2_ populations (Fig. 6). These results strongly indicate that *qRBSDV-6.3* is a genuine major QTL and can be used in future rice breeding. Thus, *qRBSDV-6.3* will be a focus for future research to mine resistance genes.

The greatest advantage of GWAS is that it can detect whether or not the tested cultivars carry favorable or unfavorable alleles at associated loci (Huang et al. 2010; Brachi et al. 2011; Zhao et al. 2011; Li et al. 2017), which is unachievable by previous linkage analysis based on double parents or a few parents. Thus, the results of GWAS can be more effectively combined with the practice of breeding. Using the information of the SNP marker loci associated with RBSDV resistance, we can select suitable parents according to needs, and achieve rapid transfer of the target loci by carrying out MAS (Li et al. 2018). Because RBSDV resistance is controlled by multiple genes, it is not practical to improve the overall RBSDV resistance level based only on marker-assisted stacking of multiple loci. It is still necessary to consciously increase the utilization frequency of resistant resources against RBSDV in traditional breeding. Our study showed that the AUS, IND and TRJ sub-populations are significantly more resistant than the ARO and TEJ sub-populations (Fig. 3). According to the RDP1 grouping by Zhao et al. (2011), most of the rice varieties grown in China belong to the IND and TEJ sub-populations. Therefore, in addition to using known resistance sources, we can appropriately increase the application frequency of cultivars in the AUS, IND and TRJ sub-populations in order to improve rice RBSDV resistance in China, especially for the TEJ sub-population rice varieties.

## Conclusions

The new resistant cultivars and QTLs against RBSDV disease identified in this study provide important information and genetic materials for the cloning of RBSDV resistance genes as well as developing RBSDV resistant varieties through marker-assisted selection.

## Methods

### Plant materials

The three hundred and five *O. sativa* accessions screened for evaluation of RBSDV resistance and used in GWAS are part of the rice RDP1, and were provided by the Genetic Stocks-Oryza (GSOR) Collection, USDA ARS Dale Bumpers National Rice Research Center, USA. They represent six major sub-populations: TRJ (76 accessions), TEJ (77 accessions), IND (59 accessions), AUS (46 accessions), ARO (6 accessions) and ADM (42 accessions) (Zhao et al. 2011; Additional file 1: Table S1). Two rice japonica cultivars with low RBSDV resistance, Wulingjing1 (WLJ-1, high susceptible) and Huaidao 5 (HD-5, high susceptible), were selected as controls for evaluation of RBSDV resistance with the RDP1 (Li et al. 2013; Wang et al. 2014).

### Evaluation of RBSDV resistance by natural infection in the field and artificial inoculation

The test locations were selected according to the occurrence of the RBSDV disease in the previous year. Experimental fields in Yutai County and Jinan City in Shandong Province (2013), Lian Yungang City (two testing locations, called lian-1 and lian-2) in Jiangsu Province (2013), and Kaifeng City in Henan Province (2014), respectively, were chosen for natural field evaluation in 2013 and 2014 due to prevalence of the RBSDV disease at these five locations in 2011 to 2013 (Zhou 2013). In 2013, the susceptible control cultivar WLJ-1 was added along each of the 40 tested varieties. In 2014, the susceptible control cultivar HD-5 was added along each of the 20 tested varieties. About 14 days before winter wheat harvest, 80 seeds per cultivar or line were sown in a 20 × 10 cm plot in the experimental field encircled by wheat plants where SBPH lived. When the wheat were harvested, SBPH moved from the wheat plants into the neighboring rice seedlings. 7 days after sowing, all seedlings were thinned to about 60 plants per line. A month later, all seedlings were moved into an experimental field. Each line was replicated three times. During the experiment, the plants were cultured with normal field management and were not sprayed with antivirals or pesticides.

Seedling individuals with typical symptoms of the RBSDV disease were considered to be susceptible plants (Ruan et al. 1984), whereas those without typical symptoms were considered resistant plants. Resistance against RBSDV was evaluated based on the RBSDV disease incidence (DI) which was calculated as the number of RBSDV-infected plants divided by the total number of plants and multiplied by 100. The survey of the incidence of the RBSDV disease was conducted at the peak tillering stage. The average DI was used for GWAS.

Artificial inoculation was performed according to the method described by Zhou et al. (2015). Non-virulent SBPH were fed on plants infected with RBSDV for 3 days to make them acquire the RBSDV. The SBPH were then transferred to rice seedlings in 5 L beakers and kept for 12 days to pass the circulative period of the virus. The random sampling and enzyme-linked immunoassay (ELISA) analysis were performed to estimate the proportion of viruliferous SBPH (Wang et al. 2006). The RBSDV viruliferous rate of SBPH was up to 32% (Additional file 8: Figure S2), indicating that there were enough virus sources. Sixty seeds per variety (three replicates) were sown into a 2 L beaker, then covered with tetoron gauze and fixed with vinyl tape. Fifty vigorous seedlings of each variety were kept for inoculation after eliminating weak seedlings at the 1.5-leaf stage. When the seedlings grow to the 2-leaf stage, 300 nymphs of RBSDV-carrying SBPH were released into each beaker. To make sure uniformity of the inoculation intensity, SBPH in each beaker were scattered three times daily for 3 days. The SBPH were subsequently removed from the dishes, and the seedlings were transplanted to a glasshouse at the Agricultural College of Yangzhou University. The RBSDV disease incidence was recorded as described in natural infection.

### GWAS analysis

GWAS analysis was performed according to the methods previously described (Kang et al. 2016), based on the publicly available 44 K-SNP data set of RDP1 accessions (Zhao et al. 2011). TASSEL 3.0 software and the mixed linear model (MLM) were used in GWAS (Bradbury et al. 2007). The MLM uses a joint kinship matrix and population structure model that can be described in Henderson’s matrix notation (Henderson 1975). To control type 1 error, regions that had more than two SNPs with *P* < 1 × 10^− 4^ within a 200-kb genomic window were considered for subsequent analysis. The Manhattan maps were plotted with PERL (Christiansen et al. 2012). *P* < 4.7 × 10^− 5^(0.0001 level) was used as the significance threshold to determine significantly associated SNP markers. EMMAX was used to fit a standard linear mixed model (Kang et al. 2010). Manhattan and quantile-quantile plots were produced by using the *R* package (https://cran.rproject.org/web/packages/qqman/).

### Introgression of *qRBSDV-6.3* through MAS

To introgress *qRBSDV-6.3* to WLJ-1/HD-5, we firstly crossed Byakkoku Y 5006 Seln / Koshihikari carrying *qRBSDV-6.3* with WLJ-1/HD-5 and then the resultant hybrid F_1_s were backcrossed with parents WLJ-1/HD-5 three times. In each generation, two flanking markers (Additional file 6: Table S6) of *qRBSDV-6.3* were deployed to select target recombinants. Finally, 4 homozygous *qRBSDV-6.3*-containing lines and 3 lines without *qRBSDV-6.3* were obtained from the Byakkoku Y 5006 Sel and WLJ-1 BC_3_F_2_ population, 6 homozygous *qRBSDV-6.3*-containing lines and 2 homozygous lines without *qRBSDV-6.3* were obtained from the Koshihikari and WLJ-1 BC_3_F_2_ population, and 2 homozygous *qRBSDV-6.3*-containing lines and 2 homozygous lines without *qRBSDV-6.3* were obtained from the Koshihikari and HD-5 BC_3_F_2_ population. The RBSDV resistance levels of these lines were evaluated in Kaifeng in 2018.

### Statistical analysis

Microsoft Excel 2010 was used to manage the data. *ANOVA* and the *Dunnett’s* multi-comparison test of the RBSDV disease scores among different varieties or sub-populations were carried out using the IBM SPSS version 16.0 (IBM Corp., Armonk, USA).

## Additional files

Additional file 1: **Table S1.** Varieties used in assay for the RBSDV disease. (DOCX 64 kb)

Additional file 2: **Table S2.** Disease incidences of the RBSDV disease and the number of SBPH landing on the 8 candidate RBSDV resistance varieties identified in 2013. (DOCX 15 kb)

Additional file 3: **Table S3.** Information of SNP markers significantly associated with RBSDV disease resistance that were identified at Kaifeng and Yutai locations. (DOCX 31 kb)

Additional file 4: **Table S4.** Information on the 32 candidate SB resistant genes in the most possible location interval (439.154 kb) of *qRBSDV-6.3*. (DOCX 20 kb)

Additional file 5: **Table S5.** Phenotype of varieties with different haplotypes formed by the five association markers. (DOCX 19 kb)

Additional file 6: **Table S6.** Primers used in this study. (DOCX 18 kb)

Additional file 7: **Figure S1.** Classification of the 32 candidate genes in the most possible location interval (439.154 kb) of *qRBSDV-6.3*. (DOCX 161 kb)

Additional file 8: **Figure S2.** RBSDV detection by enzyme-linked immunoassay (ELISA). (DOCX 78 kb)

## Data Availability

All data supporting the conclusions of this article are provided within the article (and its Additional files).

## Abbreviations

ADM: Admixture
ARO: *aromatic*
AUS: *aus*
DI: Disease incidences
ELISA: Enzyme-linked immunoassay
GWAS: Genome-wide association study
HD-5: Huaidao 5
IND: *indica*
MAS: Marker-assisted selection
MLM: Mixed linear model
QTLs: Quantitative trait loci
RBSDV: Rice black-streaked dwarf virus
RDP1: Rice diversity panel 1
RILs: Recombinant inbred lines
SBPH: Small brown planthoppers
SNPs: Single nucleotide polymorphisms
TEJ: *temperate japonica*
TRJ: *tropical japonica*
WLJ-1: Wulingjing1
