# Supplementary material for: Identification of new rice cultivars and resistance loci against rice black-streaked dwarf virus disease through genome-wide association study
Source: Rice (N Y). 2019 Jul 15;12:49. doi: 10.1186/s12284-019-0310-1 (PMC6629753; doi:10.1186/s12284-019-0310-1)
Supplement: Supplementary file 1 — Table S1. Varieties used in assay for the RBSDV disease. (DOCX 64 kb) [file 12284_2019_310_MOESM1_ESM.docx]

Additional file 1: **Table S1.** Varieties used in assay for the RBSDV disease.

| Number | NSFTV.ID | Accession.Name | Country.of.origin | Sub-population | RBSDV disease incidence in different locations | | | | |
| --- | --- | --- | --- | --- | --- | --- | --- | --- | --- |
|  |  |  |  |  | Kaifeng | Yutai | Jinan | Lian-1 | Lian-2 |
| 1 | 20 | Blue Rose | Louisiana | ADM | 10.6% | 0.0% | 0.0% | 17.2% | 21.7% |
| 2 | 40 | Dam | Thailand | ADM | 15.1% | 34.4% | 14.8% | - | 13.3% |
| 3 | 55 | Gerdeh | Iran | ADM | 96.0% | 62.5% | 33.3% | 51.6% | 44.4% |
| 4 | 87 | Keriting Tingii | Indonesia | ADM | 26.0% | 39.4% | 19.4% | 5.7% | 7.1% |
| 5 | 100 | Lacrosse | United States | ADM | 56.3% | 17.6% | 7.4% | 11.8% | 15.4% |
| 6 | 114 | Nova | United States | ADM | 58.8% | 8.3% | 5.6% | 0.0% | 5.1% |
| 7 | 128 | Pato De Gallinazo | Australia | ADM | 55.1% | 70.0% | 9.3% | 16.7% | 12.0% |
| 8 | 182 | Blue Rose Supreme | United States | ADM | 81.8% | 50.0% | 27.3% | 7.1% | 34.5% |
| 9 | 217 | YRL-1 | Australia | ADM | 52.6% | 23.8% | 4.0% | 12.9% | 22.5% |
| 10 | 218 | PI 298967-1 | Australia | ADM | 88.8% | 27.9% | 9.4% | 11.1% | 17.5% |
| 11 | 227 | Riz Local | Burkina Faso | ADM | 55.9% | 35.7% | 9.7% | 10.0% | 32.4% |
| 12 | 236 | WC 521 | China | ADM | 31.6% | 37.3% | 8.5% | 16.2% | 9.5% |
| 13 | 237 | Estrela | Colombia | ADM | 74.7% | 38.9% | 17.2% | 32.3% | 28.6% |
| 14 | 249 | Pirinae 69 | Former Yugoslavia | ADM | 100.0% | 54.2% | 19.2% | 53.3% | 16.7% |
| 15 | 253 | Guineandao | Guinea | ADM | 50.6% | 47.6% | 3.7% | 12.2% | 21.4% |
| 16 | 259 | Sadri Tor Misri | Iran | ADM | 65.8% | 22.0% | 16.1% | 16.7% | 3.8% |
| 17 | 264 | Baldo | Italy | ADM | 72.4% | 78.8% | 20.0% | 62.5% | 42.3% |
| 18 | 266 | Hiderisirazu | Japan | ADM | 43.6% | 58.1% | 24.2% | 26.9% | 47.4% |
| 19 | 270 | Osogovka | Macedonia | ADM | 78.2% | 87.5% | 45.8% | 42.1% | 55.6% |
| 20 | 273 | Varyla | Madagascar | ADM | 43.6% | 13.9% | 4.8% | 3.6% | 35.7% |
| 21 | 278 | C1-6-5-3 | Mexico | ADM | 17.5% | 26.4% | 5.9% | 8.1% | 3.6% |
| 22 | 293 | TOg 7178 | Senegal | ADM | 85.1% | 61.1% | 11.1% | 19.4% | 25.0% |
| 23 | 294 | SL 22-613 | Sierra Leone | ADM | 82.2% | 55.6% | 7.4% | 12.5% | 9.1% |
| 24 | 305 | Doble Carolina Rinaldo | Uruguay | ADM | 79.2% | 50.0% | 47.1% | 17.9% | 29.4% |
| 25 | 335 | Okshitmayin | Myanmar | ADM | 83.5% | 78.9% | 17.5% | 20.8% | 28.2% |
| 26 | 343 | Victoria F.A. | Argentina | ADM | 57.0% | 40.7% | 2.6% | 13.5% | 18.4% |
| 27 | 344 | Habiganj Boro 6 | Bangladesh | ADM | 34.2% | 75.0% | 16.0% | 20.0% | 13.3% |
| 28 | 358 | ARC 10086 | India | ADM | 58.0% | 100.0% | 35.3% | 40.6% | 33.9% |
| 29 | 364 | Rikuto Norin 21 | Japan | ADM | 48.8% | 50.0% | 12.5% | 45.7% | 24.2% |
| 30 | 367 | Sanbyang-Daeme | Korea | ADM | 67.9% | 66.7% | 15.8% | 20.0% | 22.9% |
| 31 | 376 | Breviaristata | Portugal | ADM | 100.0% | 68.8% | 7.1% | 29.0% | 30.0% |
| 32 | 386 | Palmyra | United States | ADM | 70.7% | 42.6% | 3.8% | 21.4% | 24.3% |
| 33 | 387 | M-202 | United States-CA | ADM | 65.4% | 69.6% | 14.0% | 10.3% | 38.5% |
| 34 | 388 | Nortai | United States | ADM | 45.0% | 28.0% | 31.4% | - | 16.2% |
| 35 | 389 | CI 11011 | United States | ADM | 43.0% | 11.1% | 12.5% | 10.5% | 23.4% |
| 36 | 390 | CI 11026 | United States | ADM | 53.8% | 61.2% | 17.1% | 15.9% | 1.9% |
| 37 | 622 | Bengal | United States | ADM | 44.3% | 64.3% | 13.3% | 16.7% | 31.8% |
| 38 | 627 | Early | United States | ADM | 62.0% | 38.9% | 18.8% | 18.4% | 11.4% |
| 39 | 629 | Panda | United States | ADM | 89.9% | 80.0% | 10.5% | 12.5% | 18.9% |
| 40 | 205 | Rinaldo Bersani | Italy | ADM | 69.3% | 83.3% | 27.3% | 75.0% | 52.9% |
| 41 | 271 | M. Blatec | Macedonia | ADM | 97.5% | 100.0% | 70.0% | 40.0% | - |
| 42 | 272 | 923 | Madagascar | ADM | 44.9% | - | - | - | - |
| 43 | 16 | Bico Branco | Brazil | ARO | 87.9% | 85.7% | 36.0% | 40.0% | 39.3% |
| 44 | 93 | Kitrana 508 | Madagascar | ARO | 92.9% | 61.3% | 32.4% | 22.2% | 22.2% |
| 45 | 112 | N12 | India | ARO | 93.8% | 40.0% | 6.7% | 32.1% | 21.6% |
| 46 | 160 | NSF-TV 160 | Iran | ARO | 93.8% | 33.3% | 43.2% | 22.9% | 29.4% |
| 47 | 373 | Lambayeque 1 | Peru | ARO | 100.0% | 54.5% | 19.0% | 23.3% | 42.9% |
| 48 | 124 | Pankhari 203 | India | ARO | 100.0% | 30.0% | 15.0% | 20.0% | - |
| 49 | 4 | NSF-TV 4 | India | AUS | 65.9% | 47.2% | 12.0% | 9.7% | 2.5% |
| 50 | 13 | NSF-TV 13 | Pakistan | AUS | 75.7% | 40.0% | 0.0% | 25.6% | 26.3% |
| 51 | 18 | BJ 1 | India | AUS | 48.7% | 52.8% | 5.3% | 13.9% | 0.0% |
| 52 | 19 | Black Gora | India | AUS | 30.6% | 41.7% | 8.6% | 9.4% | 14.8% |
| 53 | 44 | Dhala Shaitta | Bangladesh | AUS | 40.7% | 23.3% | 33.3% | 7.1% | 16.1% |
| 54 | 50 | DZ78 | Bangladesh | AUS | 74.7% | 84.0% | 7.7% | 26.5% | 26.4% |
| 55 | 58 | Ghati Kamma Nangarha | Afghanistan | AUS | 61.0% | 52.0% | 17.2% | - | 25.7% |
| 56 | 78 | Jhona 349 | India | AUS | 59.4% | 24.1% | 2.4% | 10.8% | 10.0% |
| 57 | 81 | Kalamkati | India | AUS | 60.5% | 35.7% | 11.1% | 2.8% | 5.6% |
| 58 | 85 | Kasalath | India | AUS | 38.5% | 46.9% | 2.9% | 10.6% | 10.3% |
| 59 | 88 | Khao Gaew | Thailand | AUS | 48.6% | 51.4% | 6.7% | 13.5% | 32.4% |
| 60 | 105 | Mehr | Iran | AUS | 48.8% | 50.0% | 11.4% | 2.7% | 3.0% |
| 61 | 131 | Phudugey | Bhutan | AUS | 48.0% | 44.0% | 0.0% | 9.7% | 11.1% |
| 62 | 152 | T 1 | India | AUS | 34.6% | 16.0% | 5.9% | 2.8% | 5.1% |
| 63 | 153 | T26 | India | AUS | 56.4% | 25.0% | 0.0% | 8.6% | 11.1% |
| 64 | 178 | ARC 6578 | India | AUS | 53.8% | 40.0% | 3.8% | 17.6% | 10.7% |
| 65 | 200 | P 737 | Pakistan | AUS | 95.4% | 66.7% | 5.9% | 32.4% | 10.8% |
| 66 | 228 | CA 902/B/2/1 | Chad | AUS | 21.3% | 15.0% | 0.0% | 0.0% | 20.0% |
| 67 | 246 | Saraya | Fiji | AUS | 22.1% | 30.2% | 3.2% | 16.2% | 0.0% |
| 68 | 261 | Shim Balte | Iraq | AUS | 80.6% | 56.3% | 8.5% | 27.5% | - |
| 69 | 262 | Halwa Gose Red | Iraq | AUS | 80.5% | 53.8% | 12.8% | 28.2% | 3.4% |
| 70 | 276 | Kaukau | Mali | AUS | 32.5% | 45.0% | 11.5% | 5.6% | 22.0% |
| 71 | 314 | CTG 1516 | Bangladesh | AUS | 66.3% | 42.9% | 6.9% | 2.8% | 10.0% |
| 72 | 316 | DD 62 | Bangladesh | AUS | 35.4% | 36.0% | 24.1% | 11.4% | 5.0% |
| 73 | 317 | DJ 123 | Bangladesh | AUS | 77.6% | 56.1% | 24.2% | 25.6% | 17.9% |
| 74 | 318 | DJ 24 | Bangladesh | AUS | 67.1% | 71.4% | 21.9% | 18.4% | 28.6% |
| 75 | 319 | DK 12 | Bangladesh | AUS | 74.7% | 44.2% | 6.1% | 13.7% | 31.7% |
| 76 | 320 | DM 43 | Bangladesh | AUS | 67.9% | 80.0% | 14.3% | 14.3% | 57.9% |
| 77 | 323 | DNJ 140 | Bangladesh | AUS | 40.5% | 57.1% | 19.4% | 11.1% | 38.5% |
| 78 | 326 | Ghorbhai | Bangladesh | AUS | 59.7% | 57.6% | 16.1% | 17.9% | - |
| 79 | 327 | Goria | Bangladesh | AUS | 12.8% | 16.3% | 3.8% | 0.0% | 7.5% |
| 80 | 328 | Jamir | Bangladesh | AUS | 39.0% | 81.3% | 51.4% | - | 9.8% |
| 81 | 330 | Khao Pahk Maw | Thailand | AUS | 45.9% | 4.8% | 0.0% | 2.6% | 0.0% |
| 82 | 331 | Khao Tot Long 227 | Thailand | AUS | 76.6% | 54.5% | 31.8% | 15.8% | 62.1% |
| 83 | 345 | DZ 193 | Bangladesh | AUS | 96.8% | 77.8% | 28.6% | 35.5% | 10.0% |
| 84 | 346 | Karkati 87 | Bangladesh | AUS | 86.5% | 50.0% | 9.1% | 18.2% | 18.2% |
| 85 | 353 | ARC 10376 | India | AUS | 35.0% | 52.8% | 3.2% | 2.6% | 3.2% |
| 86 | 357 | 9524 | India | AUS | 70.0% | 33.8% | 10.3% | 14.3% | 16.7% |
| 87 | 369 | Sathi | Pakistan | AUS | 46.8% | 55.0% | 10.3% | 14.7% | 8.8% |
| 88 | 370 | Coarse | Pakistan | AUS | 74.3% | 37.5% | 2.4% | 18.5% | 13.5% |
| 89 | 378 | Kalubala Vee | Sri Lanka | AUS | 21.5% | 10.2% | 0.0% | 0.0% | 0.0% |
| 90 | 651 | Dular | India | AUS | 26.6% | 45.0% | 10.3% | 0.0% | 0.0% |
| 91 | 49 | DV85 | Bangladesh | AUS | 71.8% | - | - | - | - |
| 92 | 312 | Aswina 330 | Bangladesh | AUS | 61.5% | 25.0% | 5.7% | 7.9% | 5.3% |
| 93 | 384 | 318 | TURKEY | AUS | 15.0% | 0.0% | 5.1% | - | 5.0% |
| 94 | 322 | DM 59 | Bangladesh | AUS | 73.4% | - | - | - | - |
| 95 | 3 | Ai-Chiao-Hong | China | IND | 9.2% | 20.5% | 5.7% | 8.3% | 10.8% |
| 96 | 17 | Binulawan | Philippines | IND | 52.1% | 57.6% | 13.0% | 23.7% | 24.3% |
| 97 | 21 | Byakkoku Y 5006 Seln | Australia | IND | 4.5% | 7.5% | 5.1% | 0.0% | 0.0% |
| 98 | 30 | Chiem Chanh | Vietnam | IND | 27.3% | 8.5% | 3.1% | 0.0% | 0.0% |
| 99 | 43 | Dee Geo Woo Gen | Taiwan | IND | 81.5% | 69.8% | 20.0% | 28.1% | 21.2% |
| 100 | 57 | NSF-TV 57 | Iran | IND | 56.0% | 20.5% | 0.0% | 3.3% | 5.6% |
| 101 | 61 | Guan-Yin-Tsan | China | IND | 51.1% | 18.3% | 3.1% | 0.0% | 8.6% |
| 102 | 71 | IR 36 | Philippines | IND | 13.8% | 34.6% | 35.3% | 5.7% | 6.3% |
| 103 | 72 | IR 8 | Philippines | IND | 24.7% | 25.0% | 17.2% | 10.8% | 7.5% |
| 104 | 74 | IRGA 409 | Brazil | IND | 54.4% | 37.0% | 10.5% | 5.3% | 20.5% |
| 105 | 76 | Jaya | India | IND | 34.6% | 36.1% | 2.2% | 18.4% | 20.5% |
| 106 | 90 | Kiang-Chou-Chiu | Taiwan | IND | 63.8% | 21.8% | 8.8% | 2.6% | 23.1% |
| 107 | 97 | Kun-Min-Tsieh-Hunan | China | IND | 8.8% | 4.5% | 0.0% | 0.0% | 0.0% |
| 108 | 106 | Ming Hui | China | IND | 25.0% | 13.0% | 1.7% | 5.9% | 2.8% |
| 109 | 109 | MTU9 | India | IND | 72.4% | 75.0% | 16.7% | 12.5% | 35.6% |
| 110 | 117 | O-Luen-Cheung | Taiwan | IND | 45.0% | 47.2% | 9.1% | 20.0% | 0.0% |
| 111 | 123 | Pagaiyahan | Taiwan | IND | 33.8% | 23.9% | 11.1% | 13.3% | 8.3% |
| 112 | 125 | Pao-Tou-Hung | China | IND | 51.3% | 48.2% | 11.4% | 10.0% | 5.0% |
| 113 | 129 | Peh-Kuh | Taiwan | IND | 100.0% | 24.0% | 8.3% | 0.0% | - |
| 114 | 130 | Peh-Kuh-Tsao-Tu | Taiwan | IND | 38.5% | 45.1% | 11.1% | 11.1% | 13.3% |
| 115 | 132 | Rathuwee | Sri Lanka | IND | 17.7% | 30.4% | 15.0% | 2.3% | 2.6% |
| 116 | 141 | Seratoes Hari | Indonesia | IND | 2.5% | 22.2% | 0.0% | 16.2% | 0.0% |
| 117 | 142 | Shai-Kuh | China | IND | 93.6% | 65.2% | 25.0% | 31.4% | 55.2% |
| 118 | 146 | Shuang-Chiang | Taiwan | IND | 41.3% | 40.0% | 3.1% | 4.2% | 6.3% |
| 119 | 172 | Zhenshan 2 | China | IND | 83.8% | 70.4% | 10.9% | 24.1% | 18.4% |
| 120 | 189 | Criollo La Fria | Venezuela | IND | 83.1% | 43.5% | 8.3% | 20.9% | 23.1% |
| 121 | 196 | JM70 | Mali | IND | 50.0% | 38.6% | 17.9% | 2.8% | 5.0% |
| 122 | 203 | Radin Ebos 33 | Malaysia | IND | 10.4% | 12.5% | 5.6% | 0.0% | 5.9% |
| 123 | 209 | Tchibanga | Gabon | IND | 84.6% | 62.5% | 9.8% | 23.4% | 37.0% |
| 124 | 222 | Paraiba Chines Nova | Brazil | IND | 28.2% | 60.0% | 2.6% | 8.6% | 17.1% |
| 125 | 231 | Hunan Early Dwarf No. | China | IND | 89.5% | 41.7% | 12.5% | 25.0% | 25.9% |
| 126 | 234 | Aijiaonante | China | IND | 82.3% | 84.2% | 22.7% | 32.3% | 28.0% |
| 127 | 235 | Sze Guen Zim | China | IND | 53.8% | 80.0% | 5.4% | 25.7% | 24.1% |
| 128 | 241 | ECIA76-S89-1 | Cuba | IND | 26.3% | 12.1% | 0.0% | 15.8% | 0.0% |
| 129 | 252 | Djimoron | Guinea | IND | 45.0% | 23.1% | 11.8% | 8.8% | 8.1% |
| 130 | 254 | Hon Chim | Hong Kong | IND | 41.0% | 24.2% | 14.3% | 0.0% | 26.7% |
| 131 | 255 | Pai Hok Glutinous | Hong Kong | IND | 48.8% | 39.3% | 6.7% | 8.8% | 9.5% |
| 132 | 284 | IR-44595 | Nepal | IND | 25.6% | 21.9% | 2.1% | 0.0% | 0.0% |
| 133 | 298 | LD 24 | Sri Lanka | IND | 30.0% | 7.4% | 6.1% | 0.0% | 11.8% |
| 134 | 299 | SML 242 | Suriname | IND | 97.4% | 75.0% | 65.5% | 5.9% | 46.2% |
| 135 | 304 | 519 | Uruguay | IND | 57.1% | 15.3% | 3.2% | 12.5% | 7.5% |
| 136 | 313 | BR24 | Bangladesh | IND | 20.5% | 42.6% | 3.2% | 21.9% | 48.7% |
| 137 | 315 | Dawebyan | Myanmar | IND | 33.3% | 50.0% | 9.1% | 11.1% | 10.0% |
| 138 | 325 | EMATA A 16-34 | Myanmar | IND | 68.0% | 57.6% | 17.5% | 33.3% | 11.8% |
| 139 | 337 | Sabharaj | Bangladesh | IND | 58.4% | 58.1% | 9.1% | 4.9% | 5.0% |
| 140 | 339 | Yodanya | Myanmar | IND | 74.7% | 60.0% | 31.7% | 26.5% | 10.7% |
| 141 | 348 | China 1039 | China | IND | 75.6% | 31.0% | 0.0% | 2.5% | 22.5% |
| 142 | 349 | Chang Ch'Sang Hsu Ta | China | IND | 69.4% | 48.8% | 34.4% | 29.0% | 23.7% |
| 143 | 356 | JC 117 | India | IND | 69.3% | 59.7% | 39.1% | 16.7% | 25.0% |
| 144 | 385 | Nira | United States | IND | 16.0% | 26.9% | 6.5% | 18.4% | 28.2% |
| 145 | 616 | RT0034 | United States | IND | 28.2% | 25.9% | 0.0% | 12.5% | 5.6% |
| 146 | 620 | Jasmine85 | Philippines | IND | 27.5% | 50.0% | 13.3% | 8.6% | 2.6% |
| 147 | 626 | C101A51 | Colombia | IND | 19.4% | 29.6% | 3.9% | 3.3% | 0.0% |
| 148 | 633 | Jing 185-7 | China | IND | 13.9% | 21.1% | 6.3% | 5.3% | 2.4% |
| 149 | 634 | Rondo (4484-1693) | China | IND | 46.2% | 14.3% | 2.0% | 0.0% | 10.5% |
| 150 | 642 | Zhenshan 97B | China | IND | 79.7% | 32.8% | 14.0% | 13.5% | 22.5% |
| 151 | 643 | Minghui 63 | China | IND | 16.3% | 5.3% | 0.0% | 5.0% | 0.0% |
| 152 | 162 | TKM6 | India | IND | 92.1% | 1.1% | 15.9% | 17.1% | 40.6% |
| 153 | 171 | ZHE 733 | China | IND | 54.4% | 75.0% | 10.7% | 33.3% | - |
| 154 | 1 | Agostano | Italy | TEJ | 100.0% | 93.8% | 11.8% | 33.3% | 0.0% |
| 155 | 9 | Baber | India | TEJ | 100.0% | 44.3% | 72.2% | 30.0% | - |
| 156 | 10 | Baghlani Nangarhar | Afghanistan | TEJ | 89.3% | 34.1% | 2.0% | 3.2% | 15.6% |
| 157 | 31 | Chinese | China | TEJ | 74.5% | 69.2% | 27.6% | 7.7% | 25.0% |
| 158 | 32 | Chodongji | South Korea | TEJ | 94.0% | 59.3% | 11.1% | 41.7% | 14.3% |
| 159 | 36 | CS-M3 | United States-CA | TEJ | 91.4% | 23.3% | 12.5% | 18.9% | 10.0% |
| 160 | 51 | Early Wataribune | Japan | TEJ | 72.9% | 63.8% | 11.4% | 38.7% | 17.9% |
| 161 | 56 | Geumobyeo | South Korea | TEJ | 67.4% | 33.3% | 25.0% | 13.5% | 15.0% |
| 162 | 64 | Heukgyeong | South Korea | TEJ | 100.0% | 67.7% | 28.6% | 21.2% | 24.3% |
| 163 | 67 | Hu Lo Tao | China | TEJ | 86.1% | 55.6% | 25.0% | 50.0% | 7.1% |
| 164 | 83 | Kamenoo | Japan | TEJ | 67.5% | 40.5% | 32.4% | 21.9% | 29.0% |
| 165 | 86 | Kaw Luyoeng | Thailand | TEJ | 44.9% | 16.7% | 12.5% | 5.7% | 7.9% |
| 166 | 91 | Kibi | Japan | TEJ | 95.0% | 71.8% | 28.1% | 18.8% | 23.3% |
| 167 | 94 | Koshihikari | Japan | TEJ | 5.0% | 6.4% | 0.0% | 2.8% | 0.0% |
| 168 | 103 | Luk Takhar | Afghanistan | TEJ | 43.8% | 28.9% | 7.5% | 8.3% | 12.5% |
| 169 | 104 | Mansaku | Japan | TEJ | 96.9% | 72.7% | 16.2% | 26.9% | 24.2% |
| 170 | 113 | Norin 20 | Japan | TEJ | 41.0% | 38.0% | 2.8% | 2.7% | - |
| 171 | 118 | Oro | Chile | TEJ | 98.7% | 81.0% | 16.0% | 35.7% | 50.0% |
| 172 | 121 | Ostiglia | Argentina | TEJ | 75.3% | 66.7% | 44.8% | 28.1% | 25.7% |
| 173 | 133 | Rikuto Kemochi | Japan | TEJ | 85.0% | 60.9% | 19.6% | 48.6% | 20.0% |
| 174 | 134 | Romeo | Italy | TEJ | 91.9% | 80.0% | 41.4% | 22.7% | 57.1% |
| 175 | 143 | Shinriki | Japan | TEJ | 90.5% | 57.1% | 29.3% | 10.3% | 29.0% |
| 176 | 144 | Shoemed | United States | TEJ | 86.8% | 58.3% | 0.0% | 2.7% | 12.8% |
| 177 | 151 | Suweon | Korea | TEJ | 67.9% | 23.8% | 10.0% | 7.7% | 15.2% |
| 178 | 154 | Ta Hung Ku | China | TEJ | 78.9% | 77.3% | 8.0% | 21.1% | 14.7% |
| 179 | 155 | Ta Mao Tsao | China | TEJ | 92.3% | 85.7% | 27.3% | 32.1% | 7.5% |
| 180 | 157 | Tainan Iku 487 | Taiwan | TEJ | 56.6% | 43.8% | 26.8% | 18.2% | 29.0% |
| 181 | 173 | Nipponbare | Japan | TEJ | 38.7% | 43.8% | 11.1% | 6.3% | 8.0% |
| 182 | 180 | Benllok | Peru | TEJ | 75.0% | 65.7% | 4.8% | 6.3% | 37.0% |
| 183 | 186 | Bul Zo | South Korea | TEJ | 81.0% | 37.2% | 32.5% | 26.3% | 26.3% |
| 184 | 219 | Nucleoryza | Austria | TEJ | 44.9% | 26.4% | 18.4% | 19.4% | 20.6% |
| 185 | 220 | Azerbaidjanica | Azerbaijan | TEJ | 100.0% | 100.0% | 60.0% | 0.0% | 50.0% |
| 186 | 224 | Karabaschak | Bulgaria | TEJ | 100.0% | 85.7% | 74.1% | 51.7% | 69.2% |
| 187 | 225 | Biser 1 | Bulgaria | TEJ | 75.9% | 87.5% | 4.1% | 26.9% | 13.3% |
| 188 | 232 | Shangyu 394 | China | TEJ | 68.4% | 62.5% | 34.2% | 18.5% | 17.5% |
| 189 | 245 | Sab Ini | Egypt | TEJ | 39.5% | 75.8% | 0.0% | 28.6% | - |
| 190 | 247 | Desvauxii | Former Soviet Unio | TEJ | 76.6% | 59.1% | 32.1% | 56.3% | 10.0% |
| 191 | 248 | Caucasica | Former Soviet Unio | TEJ | 65.0% | 48.5% | 28.6% | - | 41.4% |
| 192 | 250 | Bulgare | France | TEJ | 73.7% | 11.5% | - | 8.8% | 20.0% |
| 193 | 257 | Agusita | Hungary | TEJ | 97.2% | 0.0% | 54.2% | 33.3% | 52.2% |
| 194 | 263 | Maratelli | Italy | TEJ | 100.0% | 75.0% | 41.2% | 23.8% | 66.7% |
| 195 | 267 | Hatsunishiki | Japan | TEJ | 69.3% | - | 22.2% | 21.7% | 27.6% |
| 196 | 275 | Sri Malaysia Dua | Malaysia | TEJ | 61.8% | 68.4% | 39.5% | 4.3% | 47.4% |
| 197 | 277 | Gambiaka Sebela | Mali | TEJ | 57.7% | 44.2% | 10.4% | 31.3% | 15.0% |
| 198 | 281 | Patna | Morocco | TEJ | 35.1% | 22.7% | 19.4% | 4.3% | 2.7% |
| 199 | 282 | Triomphe Du Maroc | Morocco | TEJ | 82.1% | 60.0% | 10.2% | 17.1% | 20.0% |
| 200 | 283 | Chibica | Mozambique | TEJ | 61.3% | 48.2% | 3.8% | 16.7% | 37.5% |
| 201 | 289 | Lusitano | Portugal | TEJ | 96.3% | 25.0% | 65.2% | 13.3% | 12.5% |
| 202 | 291 | Toploea 70/76 | Romania | TEJ | 100.0% | 44.4% | 77.8% | 39.5% | 53.3% |
| 203 | 292 | Stegaru 65 | Romania | TEJ | 95.8% | 63.6% | 2.0% | 21.1% | - |
| 204 | 295 | Bombilla | Spain | TEJ | 70.1% | 65.4% | 4.3% | 23.5% | 12.5% |
| 205 | 297 | Bahia | Spain | TEJ | 68.8% | 35.7% | 25.0% | 24.2% | 26.2% |
| 206 | 300 | Sml Kapuri | Suriname | TEJ | 84.6% | 29.7% | 29.4% | 21.2% | 28.6% |
| 207 | 301 | Melanotrix | Tajikistan | TEJ | 97.3% | 100.0% | 58.6% | 60.0% | 36.4% |
| 208 | 302 | WIR 3039 | Tajikistan | TEJ | 93.4% | 82.1% | 21.1% | 28.6% | 42.4% |
| 209 | 303 | Kihogo | Tanzania | TEJ | 65.4% | 41.4% | 8.5% | 17.2% | 32.3% |
| 210 | 306 | WIR 3764 | Uzbekistan | TEJ | 94.9% | 66.7% | 54.5% | 33.3% | 28.6% |
| 211 | 307 | Uzbekskij 2 | Uzbekistan | TEJ | 97.3% | 66.7% | 41.4% | 53.8% | 50.0% |
| 212 | 311 | 56-122-23 | Thailand | TEJ | 77.5% | 51.4% | 7.3% | 19.6% | 12.5% |
| 213 | 334 | Lomello | Thailand | TEJ | 63.8% | 80.0% | 19.1% | 30.4% | 27.8% |
| 214 | 338 | Sitpwa | Myanmar | TEJ | 86.2% | 66.7% | 18.2% | 22.6% | 23.1% |
| 215 | 355 | ASD 1 | India | TEJ | 70.0% | 44.0% | 6.3% | 2.4% | 7.9% |
| 216 | 363 | Edomen Scented | Japan | TEJ | 70.7% | 75.0% | 22.2% | 8.8% | 30.0% |
| 217 | 365 | Shirogane | Japan | TEJ | 60.8% | 39.1% | 20.0% | 23.7% | 22.2% |
| 218 | 366 | Kiuki No. 46 | Japan | TEJ | 48.8% | 46.3% | 11.3% | 10.8% | 14.7% |
| 219 | 368 | Deokjeokjodo | Korea | TEJ | 100.0% | 64.5% | 22.2% | 17.6% | 20.7% |
| 220 | 380 | Tainan-Iku No. 512 | Taiwan | TEJ | 33.3% | 57.1% | 14.3% | 14.3% | 11.1% |
| 221 | 641 | Tainung 67 | Taiwan | TEJ | 30.4% | 20.6% | 0.0% | 5.0% | 5.6% |
| 222 | 79 | Jouiku 393G | Japan | TEJ | 89.9% | 37.5% | 40.0% | 25.0% | 14.3% |
| 223 | 158 | Taipei 309 | Taiwan | TEJ | 59.7% | 55.6% | 2.4% | 8.6% | 15.0% |
| 224 | 177 | 68-2 | France | TEJ | 81.5% | 66.7% | 14.3% | 21.6% | 29.2% |
| 225 | 181 | Bergreis | Austria | TEJ | 100.0% | - | - | - | - |
| 226 | 204 | Razza 77 | Italy | TEJ | 89.5% | 50.0% | 54.2% | 61.5% | 59.3% |
| 227 | 243 | Tropical Rice | Ecuador | TEJ | 223.5% | 72.7% | 71.4% | 7.9% | 50.0% |
| 228 | 265 | Vialone | Italy | TEJ | 92.5% | 88.9% | - | 33.3% | 50.0% |
| 229 | 290 | Amposta | Puerto Rico | TEJ | 62.3% | 43.8% | 22.7% | 8.7% | 13.3% |
| 230 | 216 | Yabani Montakhab 7 | Egypt | TEJ | 77.2% | - | - | - | - |
| 231 | 8 | Asse YPung | Philippines | TRJ | 16.4% | 8.3% | 9.1% | 4.3% | 8.6% |
| 232 | 14 | Basmati 217 | India | TRJ | 44.4% | 40.0% | 21.4% | 2.7% | 20.0% |
| 233 | 22 | Caawa/Fortuna 6-103-1 | Taiwan | TRJ | 74.4% | 66.7% | 18.8% | 23.8% | 33.3% |
| 234 | 24 | Carolina Gold | United States | TRJ | 10.2% | 73.3% | 3.0% | 11.1% | 23.7% |
| 235 | 25 | Carolina Gold | United States | TRJ | 42.0% | 52.9% | 18.8% | 5.9% | 25.0% |
| 236 | 26 | Carolina Gold Sel | United States | TRJ | 7.0% | 36.0% | 14.3% | 5.4% | 11.1% |
| 237 | 27 | NSF-TV 27 | Pakistan | TRJ | 100.0% | 87.5% | 5.0% | 20.5% | 14.8% |
| 238 | 37 | Cuba 65 | Cuba | TRJ | 58.8% | 23.9% | 29.2% | 5.6% | 15.0% |
| 239 | 46 | Dourado Agulha | Brazil | TRJ | 12.8% | 48.4% | 18.2% | 17.1% | 31.8% |
| 240 | 65 | Honduras | Honduras | TRJ | 63.1% | 50.0% | 30.0% | 12.9% | 14.7% |
| 241 | 69 | IAC 25 | Brazil | TRJ | 39.2% | 14.3% | 2.7% | 30.8% | 10.3% |
| 242 | 70 | Iguape Cateto | Haiti | TRJ | 84.7% | 34.6% | 18.2% | 14.6% | 33.3% |
| 243 | 73 | IRAT 177 | French Guiana | TRJ | 94.9% | 68.8% | 12.5% | 28.1% | 44.0% |
| 244 | 75 | Jambu | Indonesia | TRJ | 66.3% | 0.0% | 14.3% | 16.7% | 8.3% |
| 245 | 84 | Kaniranga | Indonesia | TRJ | 34.2% | 33.3% | 16.1% | 25.0% | 29.0% |
| 246 | 89 | NSF-TV 89 | Thailand | TRJ | 32.5% | 44.4% | 5.6% | 10.8% | 20.5% |
| 247 | 92 | Kinastano | Philippines | TRJ | 52.5% | 25.0% | 11.1% | 19.2% | 15.8% |
| 248 | 98 | L-202 | United States_CA | TRJ | 66.3% | 12.3% | 0.0% | 0.0% | 0.0% |
| 249 | 99 | LAC 23 | Liberia | TRJ | 51.9% | 44.4% | 33.3% | 0.0% | 17.9% |
| 250 | 101 | Lemont | United States | TRJ | 8.9% | 2.1% | 4.9% | 5.1% | 0.0% |
| 251 | 107 | NSF-TV 107 | Bangladesh | TRJ | 48.8% | 25.9% | 4.7% | 16.2% | 5.6% |
| 252 | 108 | Moroberekan | Guinea | TRJ | 59.0% | 28.6% | 11.8% | 13.9% | 20.7% |
| 253 | 116 | NSF-TV 116 | Pakistan | TRJ | 34.2% | 42.1% | 17.6% | 13.5% | 7.9% |
| 254 | 120 | OS6 | Nigeria | TRJ | 83.1% | 66.7% | 20.0% | 5.6% | 21.6% |
| 255 | 122 | Padi Kasalle | Indonesia | TRJ | 30.8% | 31.6% | 3.2% | 7.1% | 12.2% |
| 256 | 135 | RT 1031-69 | Zaire | TRJ | 100.0% | 0.0% | 0.0% | 8.3% | 5.0% |
| 257 | 139 | S4542A3-49B-2B12 | United States | TRJ | 82.7% | 36.4% | 25.0% | 10.0% | - |
| 258 | 147 | Sinampaga Selection | Philippines | TRJ | 61.3% | 33.3% | 0.0% | 11.4% | 13.2% |
| 259 | 149 | Sinaguing | Philippines | TRJ | 68.4% | 21.4% | 14.8% | 0.0% | 2.7% |
| 260 | 150 | Sultani | Egypt | TRJ | 90.9% | 53.8% | 8.3% | 11.4% | 24.0% |
| 261 | 164 | Tondok | Indonesia | TRJ | 86.1% | 35.0% | 42.9% | 26.3% | 28.6% |
| 262 | 165 | Trembese | Indonesia | TRJ | 93.6% | 40.0% | 44.4% | 50.0% | 25.0% |
| 263 | 167 | B6616A4-22-Bk-5-4 | United States | TRJ | 80.3% | 18.5% | 5.1% | 5.4% | 17.9% |
| 264 | 174 | Azucena | Philippines | TRJ | 50.0% | 31.3% | 15.2% | 11.1% | 17.1% |
| 265 | 176 | 583 | Ecuador | TRJ | 64.9% | 0.0% | 13.6% | 9.4% | 20.0% |
| 266 | 183 | Boa Vista | El Salvador | TRJ | 76.0% | 19.7% | 0.0% | 10.5% | 9.8% |
| 267 | 185 | British Honduras Creol | Belize | TRJ | 92.5% | 82.4% | 44.4% | 11.1% | 63.6% |
| 268 | 187 | C57-5043 | United States | TRJ | 62.2% | 23.1% | 8.2% | 8.1% | 9.4% |
| 269 | 195 | IRAT 13 | Cote D'Ivoire | TRJ | 91.9% | 42.9% | - | - | - |
| 270 | 198 | Leah | Bulgaria | TRJ | 89.9% | 54.3% | 25.0% | 62.1% | 31.0% |
| 271 | 199 | NSF-TV 199 | Bolivia | TRJ | 72.5% | 0.0% | 18.8% | 25.0% | 25.0% |
| 272 | 202 | Pratao | Brazil | TRJ | 58.4% | 21.7% | 5.0% | 20.0% | 16.7% |
| 273 | 213 | WC 3397 | Jamaica | TRJ | 41.6% | 42.1% | 18.5% | 15.4% | 2.8% |
| 274 | 215 | WC 4443 | Bolivia | TRJ | 48.8% | 26.7% | 36.4% | 29.2% | 26.7% |
| 275 | 223 | Priano Guaira | Brazil | TRJ | 44.9% | 50.0% | 8.6% | 8.6% | 11.8% |
| 276 | 229 | Niquen | Chile | TRJ | 100.0% | 50.0% | 90.9% | 33.3% | 33.3% |
| 277 | 240 | WAB 501-11-5-1 | Cote D'Ivoire | TRJ | 35.9% | 28.9% | 23.1% | 14.3% | 20.0% |
| 278 | 242 | 27 | Dominican Republic | TRJ | 25.3% | 15.4% | 15.6% | 13.9% | 25.9% |
| 279 | 251 | H256-76-1-1-1 | Argentina | TRJ | 74.4% | 35.7% | 4.8% | 2.5% | 32.0% |
| 280 | 285 | Tox 782-20-1 | Nigeria | TRJ | 45.6% | 25.0% | 7.1% | 6.5% | 8.6% |
| 281 | 286 | IITA 135 | Nigeria | TRJ | 65.4% | 34.5% | 11.9% | 11.4% | 11.1% |
| 282 | 308 | Llanero 501 | Venezuela | TRJ | 18.8% | 41.2% | 4.3% | 5.1% | 2.8% |
| 283 | 310 | R 101 | Zaire | TRJ | 94.4% | 100.0% | 63.2% | 51.9% | 50.0% |
| 284 | 352 | Guatemala 1021 | Guatemala | TRJ | 41.3% | 62.8% | 26.1% | 11.8% | 50.0% |
| 285 | 377 | PR 304 | Puerto Rico | TRJ | 21.3% | 37.0% | 11.8% | 25.0% | 11.4% |
| 286 | 381 | 325 | Taiwan | TRJ | 35.4% | 24.4% | 5.9% | 11.4% | 16.7% |
| 287 | 391 | Della | United States | TRJ | 63.8% | 42.9% | 5.0% | 7.1% | 25.0% |
| 288 | 392 | Edith | United States | TRJ | 75.9% | 50.0% | 25.0% | 12.0% | 47.1% |
| 289 | 394 | Lady Wright Seln | United States | TRJ | 73.9% | 31.3% | 2.8% | 12.8% | 12.0% |
| 290 | 395 | OS 6 (WC 10296) | Zaire | TRJ | 39.0% | 30.8% | 14.3% | 2.9% | 16.7% |
| 291 | 396 | Cocodrie | United States | TRJ | 75.0% | 30.0% | 2.0% | 23.7% | 28.9% |
| 292 | 397 | Cybonnet | United States | TRJ | 51.9% | 13.3% | 5.8% | 4.3% | 20.0% |
| 293 | 619 | Rosemont | United States | TRJ | 40.0% | 17.1% | 0.0% | 2.4% | 10.3% |
| 294 | 621 | LaGrue | United States | TRJ | 12.8% | 0.0% | 5.9% | 0.0% | 25.0% |
| 295 | 624 | Kaybonnet | United States | TRJ | 42.1% | 75.0% | 12.5% | 16.7% | 18.2% |
| 296 | 625 | Katy | United States | TRJ | 40.0% | 39.7% | 25.0% | 9.1% | 17.2% |
| 297 | 628 | Jefferson | United States | TRJ | 20.5% | 12.5% | 5.4% | 7.5% | 10.8% |
| 298 | 630 | Saber | United States | TRJ | 42.5% | 28.0% | 5.6% | 0.0% | 14.3% |
| 299 | 647 | Cypress | United States | TRJ | 45.0% | 23.5% | 1.9% | 8.5% | 19.4% |
| 300 | 54 | Fortuna | United States | TRJ | 83.7% | 17.9% | 16.7% | 41.7% | 23.3% |
| 301 | 59 | Gogo Lempuk | Indonesia | TRJ | 60.0% | 14.3% | 15.0% | - | 8.8% |
| 302 | 190 | Delrex | United States | TRJ | 86.6% | - | 31.3% | 9.1% | 0.0% |
| 303 | 226 | IRAT 44 | Burkina Faso | TRJ | 73.1% | 60.0% | 16.7% | 22.2% | 42.1% |
| 304 | 309 | Manzano | Zaire | TRJ | 85.0% | 66.7% | 12.5% | 12.5% | 18.2% |
| 305 | 239 | WAB 502-13-4-1 | Cote D'Ivoire | TRJ | 17.7% | - | - | - | - |
