# Supplementary material for: Identification of new rice cultivars and resistance loci against rice black-streaked dwarf virus disease through genome-wide association study
Source: Rice (N Y). 2019 Jul 15;12:49. doi: 10.1186/s12284-019-0310-1 (PMC6629753; doi:10.1186/s12284-019-0310-1)
Supplement: Supplementary file 2 — Table S2. Disease incidences of the RBSDV disease and the number of SBPH landing on the 8 candidate RBSDV resistance varieties identified in 2013. (DOCX 15 kb) [file 12284_2019_310_MOESM2_ESM.docx]

Additional file 2: **Table S2.** Disease incidences of the RBSDV disease and the numbers of SBPH landing on the 8 candidate RBSDV resistance varieties identified in 2013.

| **Accession Name** | **Country/**  **region of origin** | **Sub-population** | **Number of SBPH (mean±SD）** | **RBSDV disease incidence at different locations** | |
| --- | --- | --- | --- | --- | --- |
|  |  |  |  | **Kaifeng** | **Yutai** |
| Byakkoku Y 5006 Seln | Australia | IND | 14.3±3.6 | 4.5% | 7.5% |
| Chiem Chanh | Vietnam | IND | 12.7±3.2 | 27.3% | 8.5% |
| Koshihikari | Japan | TEJ | 8.4±1.5 | 5.0% | 6.4% |
| Kun-Min-Tsieh-Hunan | China | IND | 11.3±2.2 | 8.8% | 4.5% |
| Lemont | United States | TRJ | 10.5±1.7 | 8.9% | 2.1% |
| Khao Pahk Maw | Thailand | AUS | 15.7±2.2 | 45.9% | 4.8% |
| Kalubala Vee | Sri Lanka | AUS | 13.8±3.6 | 21.5% | 10.2% |
| Minghui 63 | China | IND | 12.4±2.3 | 16.3% | 5.3% |

AUS, *aus*; IND, *indica*; TEJ, *temperate japonica*; TRJ, *tropical japonica*.
