# Supplementary material for: Identification of new rice cultivars and resistance loci against rice black-streaked dwarf virus disease through genome-wide association study
Source: Rice (N Y). 2019 Jul 15;12:49. doi: 10.1186/s12284-019-0310-1 (PMC6629753; doi:10.1186/s12284-019-0310-1)
Supplement: Supplementary file 3 — Table S3. Information of SNP markers significantly associated with RBSDV disease resistance that were identified at Kaifeng and Yutai locations. (DOCX 31 kb) [file 12284_2019_310_MOESM3_ESM.docx]

Additional file 3: **Table S3.** Information of SNP markers significantly associated with RBSDV disease resistance that were identified at Kaifeng and Yutai locations.

| **SNP markers identified in Kaifeng** | | | | | **SNP markers identified in Yutai** | | | | |
| --- | --- | --- | --- | --- | --- | --- | --- | --- | --- |
| **Marker** | **Locus** | **Site** | **p** | **marker *R^2^*** | **Marker** | **Locus** | **Site** | **p** | **marker *R^2^*** |
| id1001677 | 1 | 2098423 | 3.59E-06 | 0.0692 |  |  |  |  |  |
| id1001680 | 1 | 2099423 | 3.37E-06 | 0.0695 |  |  |  |  |  |
| id1001681 | 1 | 2099471 | 3.93E-06 | 0.0684 |  |  |  |  |  |
| id1001709 | 1 | 2118023 | 5.11E-05 | 0.054 |  |  |  |  |  |
| id1001729 | 1 | 2132023 | 7.15E-05 | 0.0509 |  |  |  |  |  |
| id1001731 | 1 | 2134637 | 3.44E-05 | 0.0555 |  |  |  |  |  |
| id1001864 | 1 | 2355260 | 1.95E-05 | 0.0638 |  |  |  |  |  |
| id1002163 | 1 | 2727493 | 8.85E-06 | 0.0632 |  |  |  |  |  |
|  |  |  |  |  |  |  |  |  |  |
|  |  |  |  |  |  |  |  |  |  |
|  |  |  |  |  |  |  |  |  |  |
|  |  |  |  |  |  |  |  |  |  |
| id2007619 | 2 | 19545113 | 3.38E-05 | 0.0554 |  |  |  |  |  |
| id2007622 | 2 | 19548836 | 2.31E-05 | 0.0575 |  |  |  |  |  |
| ud3001634 | 3 | 29724621 | 3.64E-05 | 0.0559 | id3008188 | 3 | 16421475 | 9.1E-06 | 0.0649 |
| id3013976 | 3 | 29737544 | 5.56E-05 | 0.0523 | id3008208 | 3 | 16436650 | 5.4E-05 | 0.0544 |
|  |  |  |  |  | id3008281 | 3 | 16550808 | 1.7E-05 | 0.0665 |
|  |  |  |  |  | id3012226 | 3 | 27699444 | 9.2E-05 | 0.0509 |
|  |  |  |  |  | id3012227 | 3 | 27699492 | 5.7E-05 | 0.0535 |
|  |  |  |  |  | id3012850 | 3 | 28028330 | 7.1E-05 | 0.0571 |
| id4000916 | 4 | 1875972 | 1.47E-05 | 0.0708 | id4001630 | 4 | 4172738 | 2.6E-05 | 0.0644 |
| id4000919 | 4 | 1876773 | 1.18E-05 | 0.0615 | id4001777 | 4 | 4314588 | 1.8E-05 | 0.0749 |
| id4002219 | 4 | 5252695 | 9.54E-05 | 0.0494 | id4001908 | 4 | 4550573 | 9.3E-05 | 0.0583 |
| id4002220 | 4 | 5253031 | 7.55E-05 | 0.051 | id4001913 | 4 | 4551928 | 2.8E-05 | 0.0689 |
| ud4000384 | 4 | 5255769 | 4.99E-05 | 0.0623 | id4001937 | 4 | 4593387 | 3.9E-05 | 0.0598 |
|  |  |  |  |  | id4004244 | 4 | 14217795 | 3.71E-05 | 0.0597 |
|  |  |  |  |  | id4004639 | 4 | 16384319 | 0.0001 | 0.0521 |
|  |  |  |  |  | id4004640 | 4 | 16384347 | 8.5E-05 | 0.0523 |
| id6004950 | 6 | 7835229 | 9.10E-05 | 0.0498 | id6005006 | 6 | 7981536 | 1.63E-05 | 0.0845 |
| id6004955 | 6 | 7840016 | 1.69E-05 | 0.0639 | id6006808 | 6 | 10959905 | 3.86E-05 | 0.0566 |
| id6010277 | 6 | 19426454 | 4.91E-05 | 0.0544 | id6006861 | 6 | 11013833 | 1.19E-05 | 0.0637 |
| id6010459 | 6 | 19737177 | 1.45E-05 | 0.0604 | id6006865 | 6 | 11013975 | 2.51E-05 | 0.0607 |
| id6010472 | 6 | 19739735 | 3.74E-06 | 0.0694 | id6006868 | 6 | 11014079 | 5.14E-05 | 0.0599 |
| id6010489 | 6 | 19785959 | 3.02E-06 | 0.0698 | id6006918 | 6 | 11019914 | 2.52E-06 | 0.0729 |
| id6010496 | 6 | 19788314 | 3.79E-05 | 0.0558 | id6006919 | 6 | 11020282 | 1.02E-05 | 0.0687 |
| id6010523 | 6 | 19827029 | 9.64E-05 | 0.0492 | id6006937 | 6 | 11044829 | 1.84E-06 | 0.0833 |
| ud6000990 | 6 | 22715459 | 4.37E-05 | 0.0553 | id6007011 | 6 | 11101266 | 9.62E-07 | 0.082 |
|  |  |  |  |  | id6007019 | 6 | 11104110 | 4.34E-06 | 0.0715 |
|  |  |  |  |  | wd6000808 | 6 | 11106570 | 2.97E-06 | 0.0828 |
|  |  |  |  |  | id6010063 | 6 | 17993684 | 8.22E-06 | 0.0657 |
|  |  |  |  |  | id6010102 | 6 | 18088445 | 8.40E-06 | 0.0658 |
|  |  |  |  |  | id6010109 | 6 | 18114604 | 1.83E-05 | 0.0788 |
|  |  |  |  |  | id6010115 | 6 | 18116204 | 7.00E-06 | 0.0669 |
|  |  |  |  |  | id6010122 | 6 | 18119356 | 2.54E-05 | 0.059 |
|  |  |  |  |  | id6010130 | 6 | 18145976 | 4.31E-07 | 0.0895 |
|  |  |  |  |  | id6010134 | 6 | 18149332 | 8.22E-06 | 0.0657 |
|  |  |  |  |  | id6010179 | 6 | 19023715 | 3.56E-05 | 0.0595 |
|  |  |  |  |  | id6010225 | 6 | 19328416 | 4.78E-07 | 0.083 |
|  |  |  |  |  | id6010226 | 6 | 19328691 | 5.33E-07 | 0.0829 |
|  |  |  |  |  | id6010240 | 6 | 19332556 | 1.97E-06 | 0.0742 |
|  |  |  |  |  | id6010246 | 6 | 19359129 | 8.65E-06 | 0.0693 |
|  |  |  |  |  | id6010277 | 6 | 19426454 | 4.31E-08 | 0.0994 |
|  |  |  |  |  | id6010350 | 6 | 19525493 | 2.07E-06 | 0.0774 |
|  |  |  |  |  | id6010373 | 6 | 19552422 | 3.43E-07 | 0.0847 |
|  |  |  |  |  | id6010380 | 6 | 19553796 | 1.29E-06 | 0.0768 |
|  |  |  |  |  | id6010390 | 6 | 19582680 | 1.58E-07 | 0.0897 |
|  |  |  |  |  | id6010437 | 6 | 19661468 | 1.93E-07 | 0.0915 |
|  |  |  |  |  | id6010447 | 6 | 19664738 | 2.74E-07 | 0.0861 |
|  |  |  |  |  | id6010459 | 6 | 19737177 | 3.57E-05 | 0.0566 |
|  |  |  |  |  | id6010472 | 6 | 19739735 | 1.79E-05 | 0.0616 |
|  |  |  |  |  | id6010489 | 6 | 19785959 | 1.12E-05 | 0.0636 |
|  |  |  |  |  | id6010523 | 6 | 19827029 | 9.99E-05 | 0.0503 |
|  |  |  |  |  | id6010534 | 6 | 19865608 | 1.01E-05 | 0.0643 |
|  |  |  |  |  | id6010929 | 6 | 20805943 | 9.70E-05 | 0.0508 |
| id8004296 | 8 | 16332728 | 4.22E-05 | 0.0539 | id8000525 | 8 | 1787468 | 5.08E-07 | 0.1004 |
| id8004300 | 8 | 16335239 | 1.63E-05 | 0.063 | id8005330 | 8 | 19756382 | 7.39E-05 | 0.0533 |
| id8004304 | 8 | 16339623 | 1.98E-05 | 0.0592 | ud8001294 | 8 | 19756423 | 7.58E-05 | 0.0527 |
| id8004319 | 8 | 16485219 | 8.04E-05 | 0.0509 | id8005332 | 8 | 19826236 | 3.41E-05 | 0.0602 |
| id8004324 | 8 | 16485804 | 8.82E-05 | 0.0504 |  |  |  |  |  |
| id8004344 | 8 | 16524733 | 9.13E-05 | 0.0497 |  |  |  |  |  |
| id8006327 | 8 | 22038668 | 4.39E-05 | 0.0544 |  |  |  |  |  |
|  |  |  |  |  |  |  |  |  |  |
|  |  |  |  |  |  |  |  |  |  |
|  |  |  |  |  |  |  |  |  |  |
|  |  |  |  |  |  |  |  |  |  |
|  |  |  |  |  |  |  |  |  |  |
|  |  |  |  |  |  |  |  |  |  |
|  |  |  |  |  |  |  |  |  |  |
|  |  |  |  |  |  |  |  |  |  |
|  |  |  |  |  |  |  |  |  |  |
|  |  |  |  |  |  |  |  |  |  |
| id11003494 | 11 | 9028442 | 5.77E-05 | 0.0645 |  |  |  |  |  |
| id11003528 | 11 | 9075159 | 2.07E-05 | 0.0587 |  |  |  |  |  |

Note: Red markers were repeatedly detected.
