# Supplementary material for: Identification of new rice cultivars and resistance loci against rice black-streaked dwarf virus disease through genome-wide association study
Source: Rice (N Y). 2019 Jul 15;12:49. doi: 10.1186/s12284-019-0310-1 (PMC6629753; doi:10.1186/s12284-019-0310-1)
Supplement: Supplementary file 4 — Table S4. Information on the 32 candidate SB resistant genes in the most possible location interval (439.154 kb) of qRBSDV-6.3. (DOCX 20 kb) [file 12284_2019_310_MOESM4_ESM.docx]

Additional file 4: **Table S4.** Information on the 32 candidate SB resistant genes in the most possible location interval (439.154 kb) of *qRBSDV-6.3*.

| **Genes ID** | **Annotation** |
| --- | --- |
| Os06g0524300 | hypothetical protein |
| Os06g0524400 | protein trichome birefringence-like 24 |
| Os06g0524500 | cytoskeleton-associated protein 4 |
| Os06g0524700 | hypothetical protein |
| Os06g0524900 | NB-ARC domain containing protein |
| Os06g0525000 | hypothetical protein |
| Os06g0525200 | hypothetical protein |
| Os06g0526100 | transcription factor SPEECHLESS |
| Os06g0526350 | hypothetical protein |
| Os06g0526400 | abscisic acid receptor PYL3-like |
| Os06g0526466 | hypothetical protein |
| Os06g0526650 | hypothetical protein |
| Os06g0526600 | DEAD-box ATP-dependent RNA helicase 31-like |
| Os06g0526700 | probable protein phosphatase 2C 55 |
| Os06g0526800 | probable protein phosphatase 2C 56 |
| Os06g0527100 | cyclic nucleotide-gated ion channel 1 |
| Os06g0527201 | hypothetical protein |
| Os06g0527300 | cyclic nucleotide-gated ion channel 1 |
| Os06g0527500 | hypothetical protein |
| Os06g0527800 | abscisic acid receptor PYL3-like |
| Os06g0528300 | abscisic acid receptor PYL3-like |
| Os06g0528350 | hypothetical protein |
| Os06g0528600 | spermine synthase |
| Os06g0528700 | flavin-containing monooxygenase FMO GS-OX-like 9 |
| Os06g0529800 | putative E3 ubiquitin-protein ligase UBR7 |
| Os06g0529900 | Alstrom syndrome protein 1 |
| Os06g0530200 | hypothetical protein |
| Os06g0530300 | hypothetical protein |
| Os06g0530400 | NAC domain-containing protein 76-like |
| Os06g0530600 | VQ motif-containing protein 10 |
| Os06g0530700 | probable tRNA-splicing endonuclease subunit Sen2 |
| Os06g0531000 | glucan endo-1,3-beta-glucosidase 11 |
