# Supplementary material for: Identification of new rice cultivars and resistance loci against rice black-streaked dwarf virus disease through genome-wide association study
Source: Rice (N Y). 2019 Jul 15;12:49. doi: 10.1186/s12284-019-0310-1 (PMC6629753; doi:10.1186/s12284-019-0310-1)
Supplement: Supplementary file 5 — Table S5. Phenotype of varieties with different haplotypes formed by the five association markers. (DOCX 19 kb) [file 12284_2019_310_MOESM5_ESM.docx]

Additional file 5: **Table S5.** Phenotype of varieties with different haplotypes formed by the five association markers.

| Haplotype | | | | | | No. of varieties | Disease incidence at different locations (%) | | |
| --- | --- | --- | --- | --- | --- | --- | --- | --- | --- |
|  | id6010277 | id6010459 | id6010472 | id6010489 | id6010523 |  | Kaifeng | Yutai | Mean |
| 1 | A | T | C | G | T | 133 | 59.20 | 39.70 | 49.20 |
| 2 | C | A | T | A | G | 138 | 59.90 | 47.20 | 53.60 |
| 3 | A | A | T | A | G | 5 | 49.80 | 29.60 | 39.70 |
| 4 | C | A | T | A | T | 8 | 71.10 | 54.90 | 63.00 |
| 5 | C | T | C | G | T | 6 | 87.10 | 61.70 | 74.40 |
| 6 | A | T | C | G | G | 2 | 54.90 | 54.40 | 54.60 |
| 7 | C | T | C | G | G | 1 | 44.40 | 40.00 | 42.20 |
