# Supplementary material for: Identification of new rice cultivars and resistance loci against rice black-streaked dwarf virus disease through genome-wide association study
Source: Rice (N Y). 2019 Jul 15;12:49. doi: 10.1186/s12284-019-0310-1 (PMC6629753; doi:10.1186/s12284-019-0310-1)
Supplement: Supplementary file 6 — Table S6. Primers used in this study. (DOCX 18 kb) [file 12284_2019_310_MOESM6_ESM.docx]

Additional file 6: **Table S6.** Primers used in this study.

| Primers | Sequence |
| --- | --- |
| Y-19.3R | GACGGGAGTAGCCGATAT |
| Y-19.3F | AAGGGTGAGAATTGAAAGAC |
| Y-6F | AGGTGGGTGATAGGTCTC |
| Y-6R | CAAACTACTAAACGGTGC |
