# Supplementary material for: Identification of new rice cultivars and resistance loci against rice black-streaked dwarf virus disease through genome-wide association study
Source: Rice (N Y). 2019 Jul 15;12:49. doi: 10.1186/s12284-019-0310-1 (PMC6629753; doi:10.1186/s12284-019-0310-1)
Supplement: Supplementary file 7 — Figure S1. Classification of the 32 candidate genes in the most possible location interval (439.154 kb) of qRBSDV-6.3. (DOCX 161 kb) [file 12284_2019_310_MOESM7_ESM.docx]

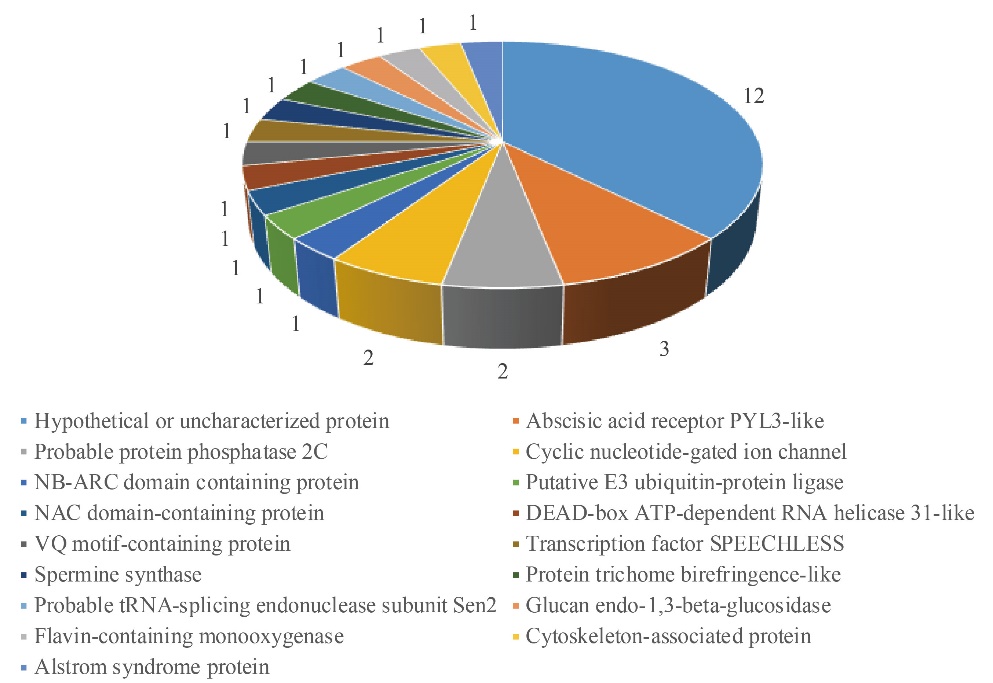


Additional file 7: **Figure S1.** Classification of the 32 candidate genes in the most possible location interval (439.154 kb) of *qRBSDV-6.3*.
