# Supplementary material for: Identification of new rice cultivars and resistance loci against rice black-streaked dwarf virus disease through genome-wide association study
Source: Rice (N Y). 2019 Jul 15;12:49. doi: 10.1186/s12284-019-0310-1 (PMC6629753; doi:10.1186/s12284-019-0310-1)
Supplement: Supplementary file 8 — Figure S2. RBSDV detection by enzyme-linked immunoassay (ELISA). (DOCX 78 kb) [file 12284_2019_310_MOESM8_ESM.docx]

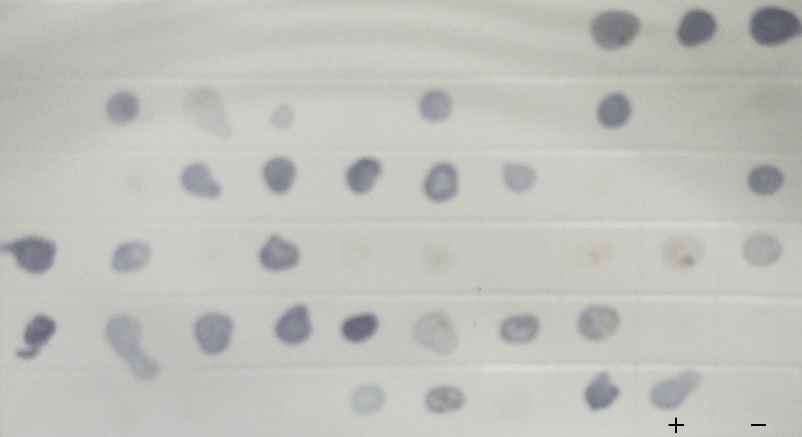


Additional file 8: **Figure S2.** RBSDV detection by enzyme-linked immunoassay (ELISA). + indicates positive control. – indicates negative control.
